# The structural basis of substrate selectivity of the acinetobactin biosynthetic adenylation domain, BasE

**DOI:** 10.1016/j.jbc.2025.108413

**Published:** 2025-03-15

**Authors:** Syed Fardin Ahmed, Andrew M. Gulick

**Affiliations:** Department of Structural Biology, University at Buffalo, Buffalo, New York, United States

**Keywords:** enzyme structure, natural product biosynthesis, siderophore, structural biology, structure-function, nonribosomal peptide synthetase

## Abstract

Siderophores are small molecule natural products that are often produced by enzymes called nonribosomal peptide synthetases that many pathogenic bacteria produce to adapt to low iron conditions. Nonribosomal peptide synthetase bioengineering could lead to the production of siderophore analogs with the potential to interrupt this unique bacterial iron uptake system, endowing the molecules with antimicrobial properties. *Acinetobacter baumannii* produces the catecholate siderophore acinetobactin to scavenge iron, a nutrient essential for several metabolic processes. Previous studies have reported synthetic analogs of acinetobactin that disrupt iron acquisition by *A*. *baumannii*, resulting in inhibition of bacterial growth. To foster a long-term goal of using a chemoenzymatic approach to produce additional analogs, we have targeted the adenylation domain BasE for the incorporation of alternate substrates. Here, we report a structure-guided approach to investigate the substrate selectivity of BasE for non-native aryl substrates. Using targeted mutagenesis in the active site of BasE, we generated mutants that catalyze the activation of alternate substrates with catalytic efficiencies comparable to the WT enzyme with its natural substrate 2,3-dihydroxybenzoic acid. We further solved structures of these mutants bound to the non-native substrates that illustrate an expanded binding pocket that support the improved promiscuity of BasE. Motivated to develop an approach to produce analogs of acinetobactin, including molecules that could block iron uptake or be readily conjugated to antibiotic cargo, our work aims to develop a structure-guided approach for using catecholate siderophore pathways to incorporate alternate substrates.

Antimicrobial resistance (AMR) is a rising global healthcare challenge that significantly affects the effectiveness of commonly used antibiotics to treat bacterial infections. The World Health Organization recently reported that in 2019, around 1.27 million global deaths were directly caused by AMR and contributed to 4.95 million deaths (1). This number is predicted to rise to 10 million by the year 2050 (1). Unfortunately, the rise in antibiotic resistance is outpacing the development of new antibiotics. The present antimicrobial development pipeline is slow and far from meeting global needs. Since 2017, only 12 new antibiotics have been developed and approved, 10 of which belong to existing classes of antimicrobials with established AMR mechanisms (2). Therefore, there is an urgent need for the development of novel antimicrobials that take advantage of nontraditional strategies for discovery and synthesis.

According to the World Health Organization list of priority pathogens, carbapenem-resistant bacteria such as *Acinetobacter baumannii* are classified as a “priority 1” pathogen for R&D of new antibiotics (3). These multidrug-resistant bacteria often produce natural products called siderophores that are high-affinity iron-chelating compounds (4). Iron is a crucial nutrient for living organisms, playing a vital role in various cellular processes, including respiration and DNA synthesis. However, in many environments, free iron is scarce and often exists in insoluble forms that are not readily accessible to microorganisms (5, 6). To overcome this challenge, many bacteria secrete siderophores that bind iron and are subsequently recognized by specific TonB-dependent receptors on the cell as a ferric complex. This complex is then transported into the cell where the iron is released and utilized for metabolic processes (7). Exploiting this unique iron acquisition strategy, siderophore-antibiotic conjugates referred to as sideromycins have been discovered naturally and further developed that use bacterial iron uptake mechanisms to deliver drugs more effectively (5, 8, 9, 10, 11, 12, 13).

Siderophores are classified into different types based on their chemical structures, with common iron-binding motifs including catecholates, hydroxamates, phenolates, and carboxylates (4, 14). Siderophores are primarily produced by two broad biosynthetic strategies, involving either nonribosomal peptide synthetases (NRPSs) or NRPS-independent siderophore synthetases. NRPSs are large, multienzyme complexes that can catalyze the biosynthesis of peptides independent of ribosomes. They function in a modular assembly line mechanism where each module consists of specialized core domains that carry out necessary functions to incorporate and modify an amino acid into the peptide (15, 16, 17, 18). These core domains include the adenylation domain (A) that selectively recognizes and activates specific amino acids, first converting them to aminoacyl-adenylate and subsequently loading them onto the pantetheine thiol group of a downstream peptidyl carrier protein (PCP) domain (19). The PCP domains, also known as the thiolation domains (T), transfer the activated amino acid to a condensation domain (C) that catalyzes a peptide bond formation between amino acid residues loaded on two sequential PCP domains (20). Once the final polypeptide is made, a terminal thioesterase domain catalyzes product release *via* cyclization (Cy) or hydrolysis (21). Additional tailoring domains, such as epimerization domains (22), methyltransferase domains (23), and Cy domains (24), are present in some modules, contributing to the structural diversity and complexity of the final peptide product (25). The modular nature of NRPSs allows for significant structural diversity in the peptide molecules they produce, which is often difficult to predict based on protein sequences. This modular nature also presents opportunities for bioengineering, where modules or domains can be recombined or altered to create novel peptides with desired properties (26, 27, 28).

A focus of NRPS bioengineering has been NRPS adenylation domains. These enzymes selectively activate amino acid residues through a two-step bi-uni-uni-bi ping pong mechanism that consists of an adenylation step and a thiolation step (29). Due to their highly specific nature, these adenylation domains play critical roles in selecting the correct amino acids to ensure the synthesis of the desired peptide product (19). Adenylation domains have two subdomains, a large N-terminal subdomain (A_core_) and a smaller C-terminal subdomain (A_sub_), which is highly dynamic, adopting unique conformations to carry out each individual step of the adenylation reaction (29). The N-terminal subdomain contains the substrate binding site, which is composed of the 10 residues that surround the pocket (30). These residues are critical for recognizing and binding the correct substrates and are typically identified through sequence and structure alignment with known adenylation domains with defined substrate specificities. The conservation of the residues that form the active site can therefore be used to predict the substrate specificity of uncharacterized adenylation domains (31). Furthermore, the understanding of the building block incorporated within an NRPS module can often be used to identify a peptide framework of a complete pathway. Together with knowledge of additional auxiliary catalytic domains, a reasonable prediction of the final NRPS product can often be made.

Previous studies have taken advantage of the highly selective nature of NRPS adenylation domains to understand the basis of its selectivity and expand its promiscuity through bioengineering. These efforts included site-directed mutagenesis, where key residues in the substrate-binding pocket were altered (32, 33, 34), and directed evolution, where large libraries of mutated adenylation domains were created and variants with desired substrate specificities were identified (35, 36). In recent years, aryl-adenylation domains such as EntE (37, 38, 39) and FbsH (40), have been engineered to both expand their substrate promiscuity and also switch their specificity code to accept non-native aryl acids. These studies demonstrated the potential of engineered adenylation domains with an expanded binding pocket to produce variants of early-stage intermediates, as well as the final products fimsbactin and enterobactin (40, 41). These non-native products were both structurally diverse and possess novel functionalities compared to the native siderophore. However, the yield for the non-native products was lower than native products, suggesting that downstream selection in the siderophore pathways could limit product yield, and that the downstream enzymes, in addition to the gatekeeper adenylation domains, have potential for engineering. Recent studies have used a directed evolution approach to engineer the enterobactin biosynthetic enzyme EntF to alter its adenylation domain substrate selectivity (42). Using a chimeric EntF consisting of a noncognate adenylation domain, coupled with the use of mutant libraries, several substrate analogs were incorporated into the pathway to produce novel catecholate products. Finally, recent studies have also engineered terminal adenylation domains, to generate functionalized derivatives of siderophores *in vivo* (43). These studies demonstrate the significance of rational engineering of NRPS adenylation domains, to produce novel, physiologically relevant siderophore analogs and the ability of downstream domains to accommodate newly introduced substrate analogs, although often with a decrease in pathway throughput.

The catecholate siderophore acinetobactin, produced by *A. baumannii*, has been a key focus of study for the generation of functionalized derivatives. The biosynthetic mechanism of acinetobactin assembly was first proposed based on a homologous pseudomonine biosynthetic pathway due to significant similarity between their NRPS gene clusters (25, 44). The biosynthesis begins with BasE, a standalone adenylation domain, which activates and loads 2,3-dihydroxybenzoic acid (DHB) to the phosphopantetheinyl arm of the PCP domain of BasF (Fig. 1). Simultaneously, the PCP domain of BasB is loaded with L-threonine by BasA, another adenylating enzyme. An amide bond is then formed between DHB and L-threonine by BasD, followed by cyclodehydration resulting in a catechol oxazoline intermediate, loaded on BasB. As BasD consists of two Cy domains, the precise order of the Cy domains actions remains unclear. However, by analogy with the tandem Cy domains of vibriobactin (45) and fimsbactin (46), it is predicted that Cy2 is likely to perform amide bond formation, whereas Cy1 likely catalyzes cyclodehydration. Finally, the condensation domain of BasB facilitates a nucleophilic attack of *N*-hydroxyhistamine on the oxazoline intermediate, leading to the formation of preacinetobactin, which can then isomerize to form acinetobactin (47).

**Figure 1:**
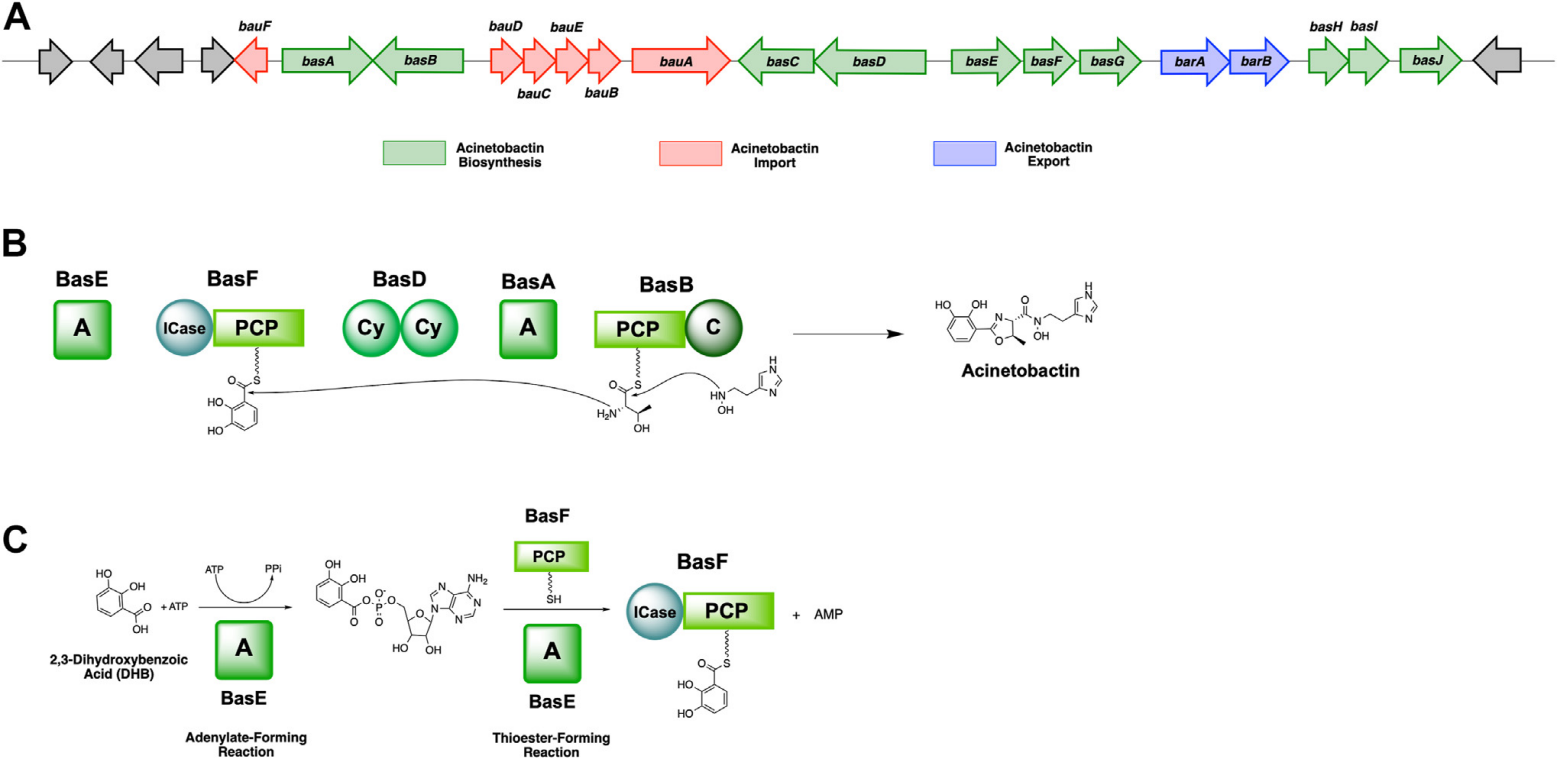
**Biosynthesis of acinetobactin.***A*, biosynthetic gene cluster for acinetobactin biosynthesis from *Acinetobacter baumannii* AB900 encodes proteins involved in biosynthesis (*green*), import (*red*), and export (*blue*). *B*, the biosynthesis of acinetobactin employs the NRPS proteins BasABDEF. Adenylation domains of BasE and BasA load DHB and threonine, respectively, which are combined through the activities of the cyclization domains of BasD. The condensation domain of BasB catalyzes transfer to an *N*-hydroxyhistamine. *C*, two-step adenylation reaction catalyzed by the standalone adenylation domain BasE. Domains labels. A, adenylations; C, condensation; Cy, cyclization; ICase, isochorismatase; NRPS, nonribosomal peptide synthetase; PCP, aryl/peptidyl carrier protein.

Synthesis of acinetobactin analogs has been performed to identify critical regions for iron binding and transport. The thiazoline analog anguibactin, built from a cysteine rather than serine substrate, has also been produced along with its salicylate counterpart deoxyanguibactin (48). These thermally stable molecules bind iron and support growth of *A. baumannii*. Additionally, a panel of compounds has been explored with modifications at the hydroxamate and histamine groups (49). Given the role of acinetobactin in virulence (50), analogs have also been produced that disrupt the iron uptake pathways. Synthetic analogs of acinetobactin with alternate connectivity of the iron-binding groups or that oxidize the central oxazoline ring to rigidify the structure of acinetobactin are potent inhibitors of siderophore transport, inhibiting the growth of *A. baumannii* (51, 52, 53). A larger modification to the aryl ring was reported with 5-phenyl-preacinetobactin, which retains the ability to bind iron but fails to rescue growth; here, it was not tested if the molecule actually competed with natural acinetobactin for iron uptake (54). Compounds have also been described that block the biosynthesis of acinetobactin by inhibiting the gatekeeper adenylation domain BasE (55, 56), raising the possibility that analogs of acinetobactin might be useful as biosynthetic inhibitors. While chemical synthesis of acinetobactin analogs has enabled these studies, a highly efficient chemoenzymatic strategy for the biosynthesis of novel acinetobactin analogs with antimicrobial properties has yet to be developed.

Toward a long-term biocatalytic approach to produce siderophore analogs, we expand on previous studies with aryl adenylating enzymes by exploring the substrate selectivity of BasE and use targeted engineering to expand its promiscuity for non-native aryl acids. Using structure-guided mutagenesis, we engineer several binding pocket residues to generate variants of BasE and screen for substrate specificity. We focus our attention specifically on aryl acids with modifications at the C4 position, requiring changes to the base of the substrate-binding pocket. This leaves intact the C2 hydroxyl that is required for iron binding. Through steady-state kinetic analysis of select mutants, we show that our targeted engineering significantly improves the catalytic efficiencies of BasE for substrate analogs, resulting in mutant enzymes that are comparable with alternate substrates to that of WT BasE with its native substrate DHB. Finally, we present several substrate-bound structures of BasE variants and provide evidence that the improvement observed in catalytic efficiencies for DHB analogs are a direct result of our targeted protein engineering. Overall, our work establishes the foundations for the discovery of novel acinetobactin analogs with antimicrobial properties, through chemoenzymatic approaches using our designed variants.

## Results

### Enzymatic activity of BasE with DHB analogs

**Figure 2:**
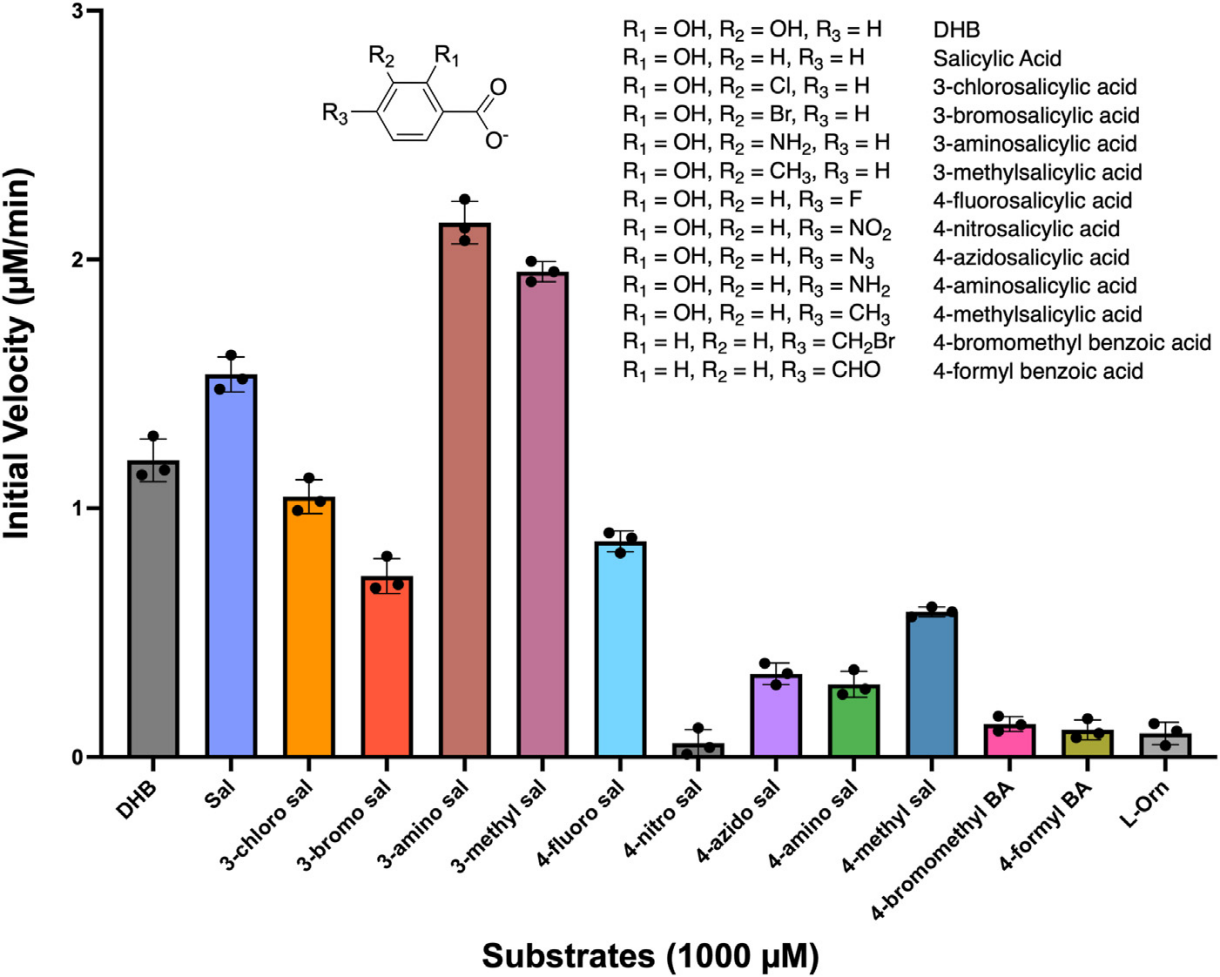
**Activity profile of WT BasE with substrate analogs.** Specific activity screening of WT BasE using measurement of initial velocities showed an overall preference for salicylic acid and C3-substituted analogs over analogs containing a constituent at C4. L-Ornithine was used as a negative control. Data are presented as mean ± SD with assay triplicates superimposed.

To gain insight into substrate selectivity of BasE for DHB analogs, we first investigated its catalytic activity with a panel of substrates. These analogs included salicylic acid that lacks the C3 position hydroxyl group and derivatives with different constituents at the C3 and C4 positions. We began by assessing substrate selectivity through measuring initial velocities with a range of these substrate analogs, using an AMP detection assay that couples AMP formation to NADH oxidation (Fig. S1). BasE exhibited comparable activities with both DHB and salicylic acid, indicating that removal of the hydroxyl group from the C3 position has minimal effect on substrate adenylation (Fig. 2). Results with other analogs indicated that WT BasE prefers C3-substituted analogs over those with C4 substitutions, likely due to spatial constraints at the base of the DHB-binding pocket. Notably, several C4-substituted analogs, including 4-nitrosalicylic acid and 4-formyl benzoic acid, showed very minimal activity.

**Figure 3:**
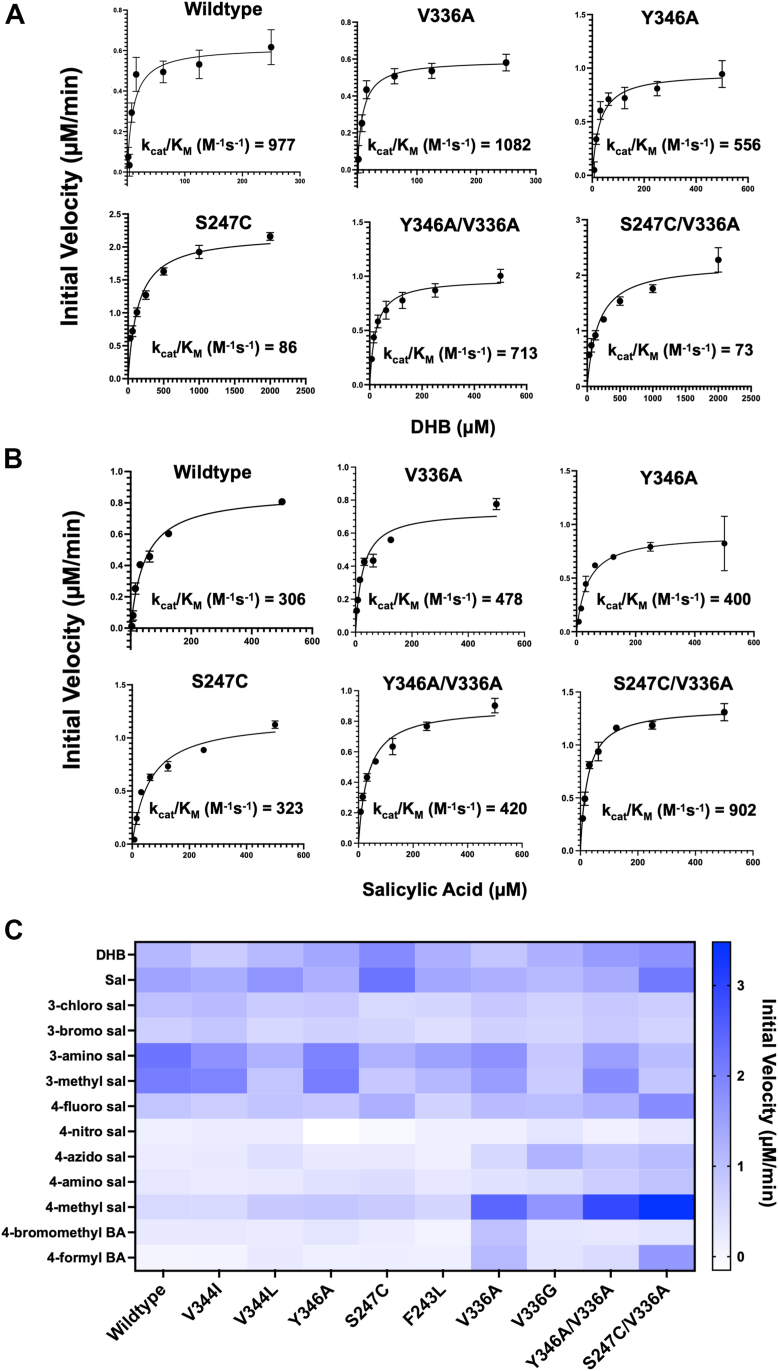
**Activity profile of mutant BasE with substrate analogs.** Steady-state kinetic analysis of WT and select mutant BasE with the native substrate DHB (*A*) and salicylic acid (*B*). The BasE mutants retain activity for DHB although the S247C and S247C/V336A mutants switch substrate specificity to prefer salicylic acid. *C*, heat map representing initial velocity of WT and mutant BasE enzymes with substrate analogs. Data are presented as average of triplicates. DHB, 2,3-dihydroxybenzoic acid.

To obtain a better understanding of enzymatic activity, we conducted steady-state kinetic analysis that allowed us to evaluate the binding affinity and catalytic efficiency of BasE with the numerous analogs. We first compared the apparent catalytic efficiencies of DHB (Fig. 3*A*) and salicylic acid (Fig. 3*B*) to verify whether they were consistent with our findings from the specificity screening. Steady-state kinetic analysis showed that the catalytic efficiency of WT BasE with salicylic acid is 3-fold lower than that of DHB, primarily due to a lower binding affinity reflected in the apparent K_M_ (Table 1), likely attributable to the lack of hydrogen bonding between the C3 hydroxyl group and Asn242 and Ser247. We next conducted kinetic analysis with additional substrate analogs. We focused on C4-substituted analogs as our goal was to expand the selectivity of BasE for these analogs using rational engineering. The apparent kinetic constants of C4-substituted analogs exhibited catalytic efficiencies that were around 40 to 140 times lower than DHB. This reduction was also attributed to diminished binding affinities (apparent K_M_), with C4-substituted analogs showing values between 650 and 740 μM, compared to 10.5 ± 2.8 μM for DHB. Specifically, 4-fluorosalicylic acid demonstrated roughly 40-fold reduction in catalytic efficiency, while 4-methylsalicylic acid showed over a 140-fold decrease compared to DHB (Fig. S2).

**Table 1:** Apparent steady-state kinetics of WT and mutant BasE with different aryl substrates

Protein	Substrate	[Protein] (μM)	k_cat_ (min^−1^)	K_M_ (μM)	k_cat_/K_M_ (M^−1^s^−1^)	Relative activity^a^
WT	DHB	1	0.62 ± 0.04	10.5 ± 2.8	977	1.00
V336A	DHB	1	0.59 ± 0.02	9.2 ± 1.7	1080	1.11
V336G	DHB	1	0.66 ± 0.06	20.8 ± 6.5	528	0.54
Y346A	DHB	1	0.96 ± 0.05	28.8 ± 6.2	556	0.57
S247C	DHB	3	0.73 ± 0.02	142 ± 16	86	0.08
V336A/Y346A	DHB	1	0.98 ± 0.03	22.9 ± 2.9	713	0.73
V336A/S247C	DHB	3	0.74 ± 0.04	169 ± 29	73	0.07
WT	Sal	1	0.86 ± 0.03	47.0 ± 5.5	306	1.00
V336A	Sal	1	0.74 ± 0.03	25.7 ± 3.9	478	1.56
V336G	Sal	1	0.92 ± 0.10	103 ± 32	148	0.48
Y346A	Sal	1	0.91 ± 0.05	37.9 ± 7.8	400	1.31
S247C	Sal	1	1.18 ± 0.05	60.9 ± 8.3	323	1.06
V336A/Y346A	Sal	1	0.90 ± 0.03	35.6 ± 4.0	420	1.37
V336A/S247C	Sal	1	1.36 ± 0.03	25.0 ± 2.1	902	2.95
WT	4-fluoro sal	1	0.97 ± 0.10	653 ± 131	25	1.00
V336A	4-fluoro sal	1	0.68 ± 0.04	35.6 ± 7.5	321	13.1
V336G	4-fluoro sal	1	0.47 ± 0.04	37.6 ± 15.2	209	8.5
Y346A	4-fluoro sal	3	0.39 ± 0.01	213 ± 27	30	1.24
S247C	4-fluoro sal	3	0.46 ± 0.02	225 ± 39	34	1.39
V336A/Y346A	4-fluoro sal	0.5	1.92 ± 0.17	66.3 ± 18	484	19.7
V336A/S247C	4-fluoro sal	1	1.83 ± 0.06	32.1 ± 4.2	953	38.7
WT	4-methyl sal	5	0.30 ± 0.03	731 ± 165	6.9	1.00
V336A	4-methyl sal	1	1.47 ± 0.04	46.5 ± 4.62	527	76.1
V336G	4-methyl sal	1	0.91 ± 0.04	24.6 ± 4.71	617	89
Y346A	4-methyl sal	3	0.64 ± 0.07	902 ± 203	11.7	1.69
S247C	4-methyl sal	3	0.62 ± 0.07	1420 ± 320	7.3	1.06
V336A/Y346A	4-methyl sal	1	1.61 ± 0.04	55.8 ± 4.3	480	69.3
V336A/S247C	4-methyl sal	1	3.55 ± 0.18	86.8 ± 13	682	98.4
WT	4-azido sal	--	--	--	--	--
V336A	4-azido sal	3	0.29 ± 0.03	674 ± 150	7.1	1.86
V336G	4-azido sal	1	0.61 ± 0.05	67.4 ± 19	151	39.5
Y346A	4-azido sal	--	--	--	--	--
S247C	4-azido sal	3	0.10 ± 0.01	428 ± 112	3.8	--
V336A/Y346A	4-azido sal	1	1.11 ± 0.12	564 ± 126	32.7	8.56
V336A/S247C	4-azido sal	3	0.27 ± 0.02	181 ± 51.9	25.2	6.60

DHB, 2,3-dihydroxybenzoic acid.

a Relative activity is the ratio of catalytic efficiency (k_cat_/K_M_) for a mutant enzyme compared to WT enzyme with the same substrate. For 4-azidosalicylic acid, relative rates are presented compared to the S247C mutant.

### Structure-guided mutagenesis of BasE to expand selectivity for analogs

We used the structure of WT BasE (55) as a guide to alter specific binding pocket residues to expand its substrate selectivity for DHB analogs, particularly the C4-substituted DHB analogs that had minimal activity with WT BasE. Several binding pocket residues were identified within 5 Å of the ligand, including Asn242, Phe243, Ser247, Val336, Val344, and Tyr346. While Ser247 and Asn242 formed hydrogen bonds with the hydroxyl groups of DHB, the remaining four residues formed the hydrophobic base of the pocket. In the structure of BasE bound to DHB-AMS (PDB 3O82) (55), the C4 carbon of the DHB is positioned 3.6 Å from the Cγ of Val336, 4.2 Å from the Cβ of Ser247, 4.4 Å from the Cγ of Val344, and 3.6 Å from the Cβ of Phe243 which suggested that steric clashes may hinder the ability of the C4-substituted substrates from adopting a proper position for efficient binding and adenylation. To accommodate C4-substituted analogs, we generated four mutants, V336A, V336G, Y346A, and F243L. Tyr346, located in the secondary shell of the binding pocket, and 6.5 Å from the C4 carbon of DHB, was targeted to accommodate even larger 4-substituted analogs such as 4-azidosalicylic acid.

The equivalent residue to Ser247 has been identified as an important determinant of DHB or salicylate binding specificity initially from structural studies (57) and subsequently in biochemical tests with EntE (38), where the S240C mutant has a 10-fold lower K_M_ value with salicylic acid than DHB. It appears that the larger, delocalized thiol encroaches on space that is adopted by the C3 hydroxyl of DHB. As most of our analogs were salicylic acid analogs that lacked the C3 position hydroxyl group, we also tested whether mutating the binding pocket residues to mimic the homologous salicylic acid binding adenylation domain YbtE (58) could expand its activity for these analogs. Therefore, we engineered several mutations for this purpose which included S247C, V344I, and V344L. Finally, in addition to single mutants, we also created double mutants S247C/V336A and Y346A/V336A to test whether single mutants together could have a combined effect of further expanding activity for substrate analogs. These mutant enzymes were used both for activity assays as well as for structural studies (see below) to explore their ability to activate alternate substrates.

All nine mutants of BasE were next screened for specific activity with the same panel of substrates that were tested with WT (Fig. 3*C*). For the C3-substituted analogs, most mutants either had equivalent or lower activity than WT, suggesting that the WT residues could be forming important hydrophobic interactions with these specific analogs. For the C4-substituted analogs, four mutants (which included the double mutants) that expanded the binding pocket at the C4 position of DHB showed improvement in specific activity. Therefore, we focused on these enzyme variants to conduct steady-state kinetic analysis to further validate our activity profile.

### Steady-state kinetic analysis of BasE mutants

We conducted steady-state kinetic analysis with promising mutants and the panel of substrates (Table 1). In addition to analyzing the WT enzyme, we selectively screened six mutants of BasE that had shown enhancement in activity in our specificity screening. These include the single mutants V336A, V336G, Y346A, and S247C as well as the double mutants V336A/Y346A and V336A/S247C. For the native substrate DHB and salicylic acid, the mutants retained their comparable activities with the WT enzyme (Fig. 3 and Table 1). The mutant S247C (Table 1 and Fig. S3) and the double mutant S247C/V336A showed reduced catalytic efficiency for DHB by more than 10-fold. The double mutant had significantly higher activity for salicylic acid (∼3-fold) emphasizing the importance of the hydrogen bond formation between the Ser247 and the C3 position hydroxyl group of DHB.

We next assayed the C4-substituted analogs of salicylic acid with both the WT and mutant enzymes. Consistent with what we observed in our specificity screening experiments, several of the engineered mutants had significantly higher catalytic efficiencies for the C4-substituted analogs than the WT enzyme. The V336A mutation (Table 1, Supplemental Fig. S4) that expands the binding pocket of BasE near the C4 position of DHB, resulted in a ∼13-fold improvement in apparent k_cat_/K_M_ for 4-fluorosalicylic acid and a ∼76-fold improvement for 4-methylsalicylic acid. The V336G mutation (Table 1 and Fig. S5) further expands the binding pocket, showing improved activity for both 4-fluorosalicylic acid (∼9 fold) and 4-methylsalicylic acid (∼89 fold). This mutant also had significantly higher apparent k_cat_/K_M_ for 4-azidosalicylic acid (∼40 fold), a trend that was not observed for several of our other mutants. The Y346A mutation in isolation had very limited impact on the catalytic efficiency with the alternate substrates (Table 1 and Fig. S6). However, the double mutant V336A/Y346A (Table 1 and Fig. S7), where both the primary and the secondary shell of the binding pocket was mutated to expand the pocket at the base, had a ∼69-fold improvement in activity for 4-methylsalicylic and a ∼20-fold improvement for 4-fluorosalicylic acid (Table 1). Finally, the most dramatic improvements in catalytic efficiency were observed for the double mutant V336A/S247C, which had a ∼40-fold improvement for 4-fluorosalicylic acid and a ∼100 fold improvement for 4-methylsalicylic acid (Table 1 and Fig. S8). The activities of this double mutant with 4-fluorosalicylic acid and 4-methylsalicylic acid were comparable to that of WT BasE with its native substrate DHB. These improvements in catalytic efficiencies were mainly attributed to the significant reduction in apparent K_M_ observed with the mutants indicating that the mutagenesis likely improved the binding affinities of these substrates.

### Structural analysis of BasE mutants bound to substrate analogs

**Figure 4:**
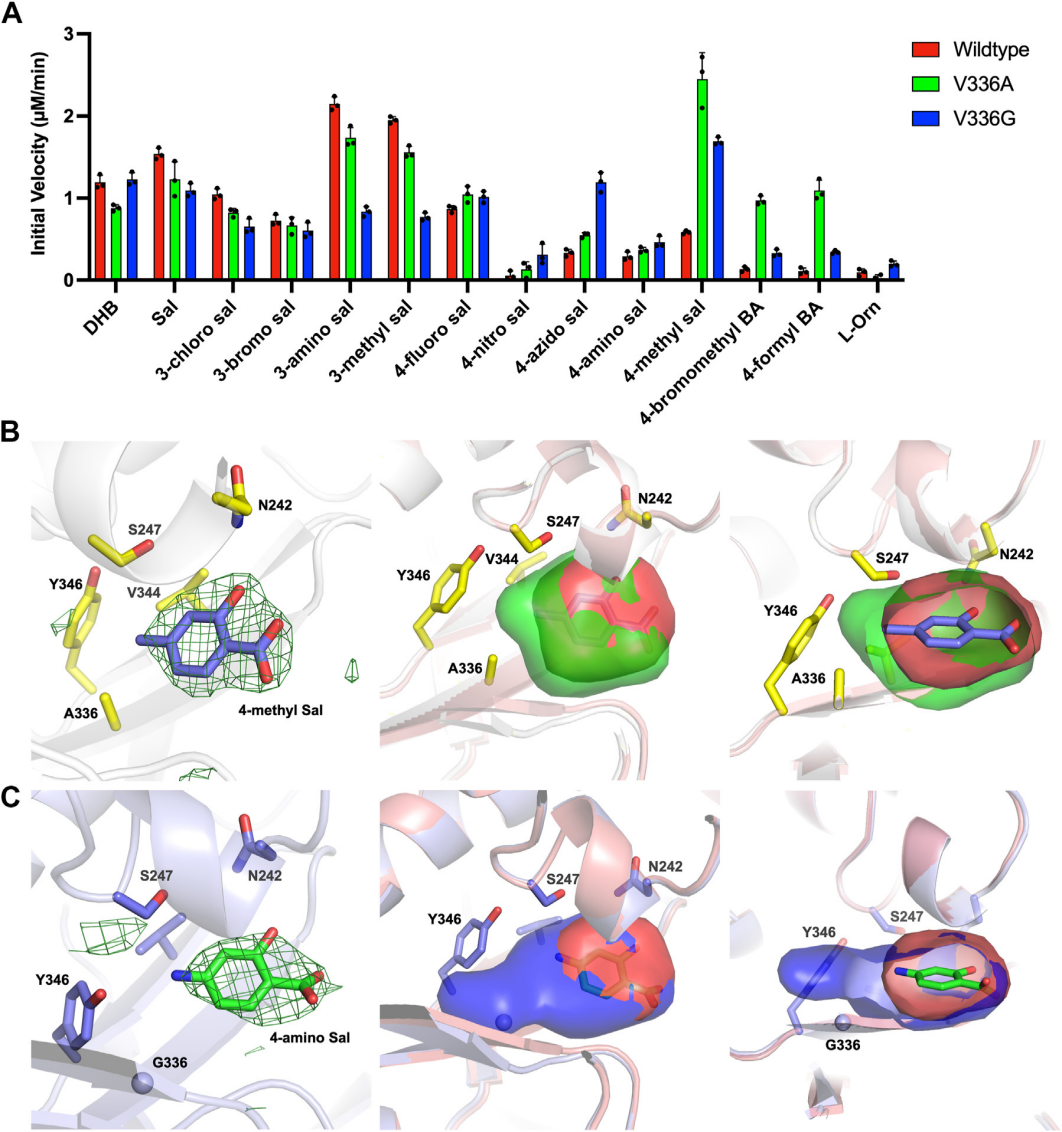
**Specificity screening and structural analysis of the BasE mutants V336A and V336G.***A*, the two mutations expand activity for 4-substituted salicylic acid analogs. Data are presented as mean ± SD with assay triplicates superimposed. *B*, the V336A mutant bound to 4-methylsalicylic acid had an expanded binding pocket (*green*) compared to WT BasE (*red*) that could accommodate larger substrate analogs. *C*, the V336G mutant (*blue*) had a further expanded pocket compared to both WT and V336A. Simulated annealing omit map density was calculated by removing ligand from the final model and refining with simulated annealing. Density is shown with coefficients of the form Fo-Fc, contoured at 3σ with a carve distance of 5 Å. The casting of pocket volumes of WT and mutant enzymes were generated using HOLLOW.

**Figure 5:**
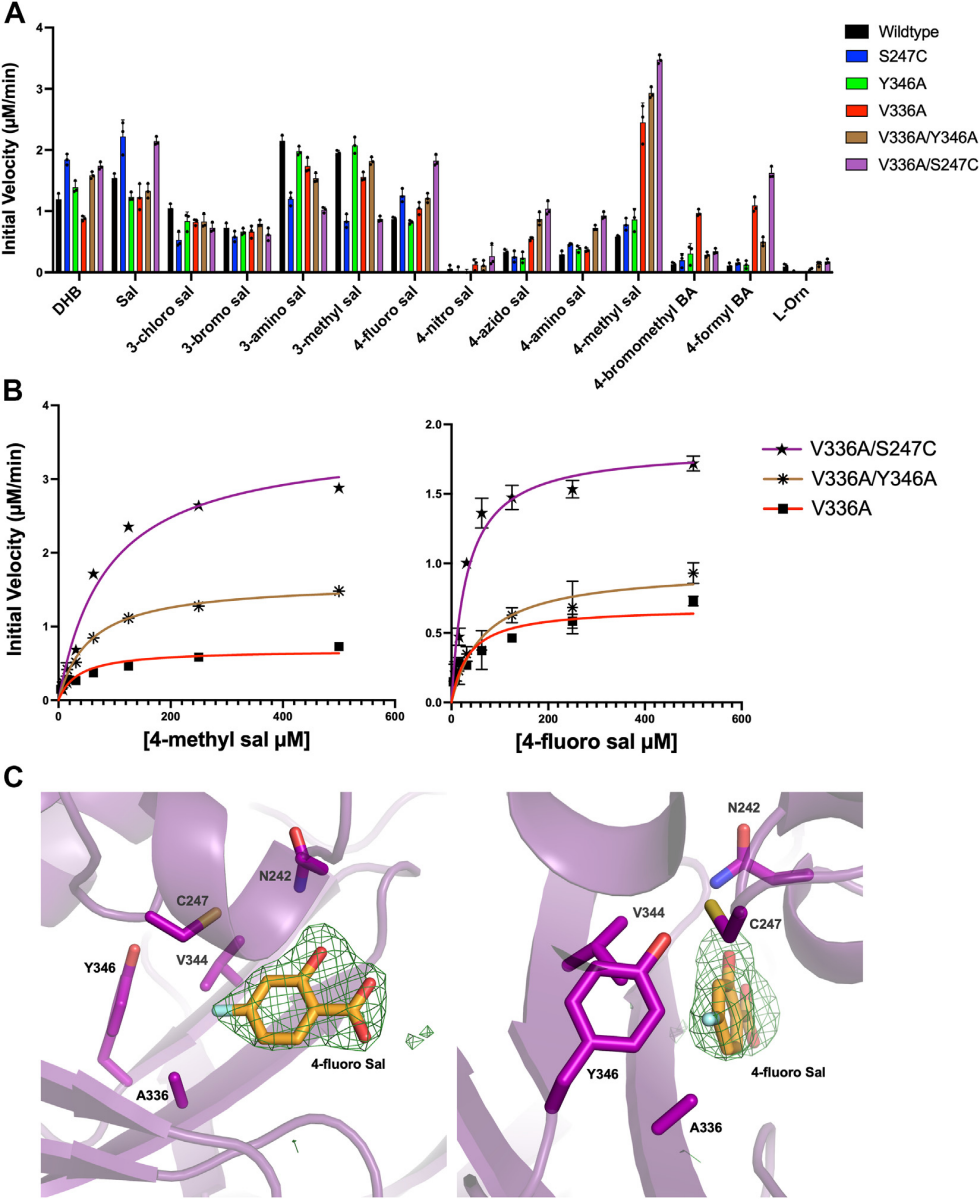
**Specificity screening and steady-state kinetic analysis of BasE single mutants compared to double mutants.***A*, double mutants of BasE had higher specific activity for several C4-substituted substrate analogs than both WT and single mutants. Data are presented as mean ± SD with assay triplicates superimposed. *B*, steady-state kinetic analysis showed that the V336A/S247C double mutant had the highest activity for several substrate analogs with a ∼39-fold improvement for 4-fluorosalicylic acid and a ∼98-fold improvement for 4-methylsalicylic acid. This catalytic activity was comparable to WT BasE with its native substrate DHB (Table 1). *C*, structure of V336A/S247C bound to 4-fluorosalicylic acid. Simulated annealing omit map density was calculated by removing ligand from the final model and refining with simulated annealing. Density is shown with coefficients of the form Fo-Fc, contoured at 3σ with a carve distance of 5 Å. DHB, 2,3-dihydroxybenzoic acid.

We solved the structures of the V336A and V336G mutants, which showed a general improvement in activity with the C4-substituted substrates from our panel (Fig. 4*A*) and the structure of the V336A/S247C double mutant, which showed some of the highest activities among our mutants (Fig. 5*A*) bound to 4-fluorosalicylic acid. All three structures were solved in the P2_1_2_1_2_1_ with two molecules per asymmetric unit. The V336A structure was first solved using the N-terminal subdomain of the WT structure of BasE (3O83) (55) as the molecular replacement search model. No density was observed for the dynamic C-terminal subdomain, as observed in the prior BasE structures. Next, using the protein atoms from the refined structure of V336A as a search model, the structures of V336G and V336A/S247C were solved. Ligands were added to all three structures at late stages of refinement, after manual model building and refinement of protein atoms and solvent molecules. Although the V336G BasE enzyme was soaked with 4-azidosalicylic acid, weak density was observed for the azido group of this molecule. As the azido group may be flexibly positioned or has the potential to hydrolyze to an amino group, possibly promoted by the reducing agent tris(2-carboxyethyl)phosphine (TCEP) (59), we placed 4-aminosalicylic acid in the observed density, which fit well (Fig. 4*C*). We docked the 4-azidosalicylic acid molecule into our V336G structure, which showed that the ligand has the potential to bind similarly as our expected pose, with the azido group pointing toward the V336G mutation (Fig. S9).

The V336A mutant bound to 4-methylsalicylic acid (Fig. 4*B*) and the V336G mutant bound to 4-aminosalicylic acid (Fig. 4*C*) were used to compare with the binding pocket volume of the WT enzyme. To assess the expansion of the binding pocket in our mutant BasE structures, the software package HOLLOW was utilized. We first generated a casting of the binding pocket of WT BasE and compared its volume to V336A (Fig. 4*B*). We observed a significant expansion of the pocket near the region where the valine to alanine mutation was introduced, suggesting that the methyl group of 4-methylsalicylic acid was better coordinated into the mutant binding pocket as a result of this extra space. The binding pocket of V366G showed an even larger expansion (Fig. 4*C*), which provided further space for the azido group of 4-azidosalicylic acid, resulting in the near 40-fold improvement in activity compared to the V336A mutant. When comparing V336G structure to the other BasE structures, we also observed the nearby Tyr346 to slightly turn toward Gly336 as a result of the extra space being present (Fig. S10). However, the conformations of the remaining binding pocket residues were maintained.

We then analyzed the structure of the double mutant V336A/S247C, which exhibited both an increase in space in the binding pocket and the introduction of the cysteine residue observed in aryl adenylating enzymes that bind salicylate. This mutant demonstrated some of the highest increases in specific activity (Fig. 5*A*) and showed improved catalytic efficiencies with the 4-substituted analogs, comparable to WT BasE for DHB (Fig. 5*B* and Table 1). This structure supported the limited changes in the pocket that resulted, however, in increased space to accommodate the larger analogs at the base of the pocket. Overall, our structural analysis using ligand-bound structures of BasE mutants provided evidence of binding pocket expansion and further validated our kinetic analysis on the enhancement in activity of BasE mutants for substrate analogs.

## Discussion

In this study, we expand on previous work of NRPS adenylation domains from our lab (40) and others (32, 41, 42) to understand the substrate selectivity of aryl adenylating enzymes involved in siderophore biosynthesis. Using rational site-directed mutagenesis, we generated nine mutants of BasE and tested their selectivity with a panel of substrate analogs. We showed that our engineered mutants enhanced the ability of BasE to recognize non-native substrates and identified determinants of its substrate selectivity for alternate substrates. Previous studies have engineered homologous adenylation domains like EntE to better incorporate C2- and C3-substituted analogs of benzoic acid (37, 39, 41). However, analogs that affect the C2 hydroxyl may result in products with reduced ability to bind iron. Our study aimed to expand the selectivity of BasE to incorporate analogs that would retain the ability of acinetobactin to bind iron and subsequently bind to siderophore-binding proteins. These included C4-substituted analogs of salicylic acid, which contain the C2 position hydroxyl group necessary for iron chelation and delivery but lack the C3 position hydroxyl group that has been shown to be dispensable in Acb-Oxa analogs (49, 51). Our data suggest that an expanded binding pocket enables BasE to accommodate these larger C4-substituted substrate analogs of DHB. When combined with mutations that switch the specificity code of BasE to prefer salicylic acid, this activity can be further improved and be comparable to the activity of the WT enzyme with its native substrate DHB. We further solved structures of several of these engineered mutants each bound to unique substrates that had significantly improved activities. Our data support our previous work with a homologous adenylation domain FbsH as well as other well characterized homologous adenylation domains like EntE (37, 38, 39).

**Figure 6:**
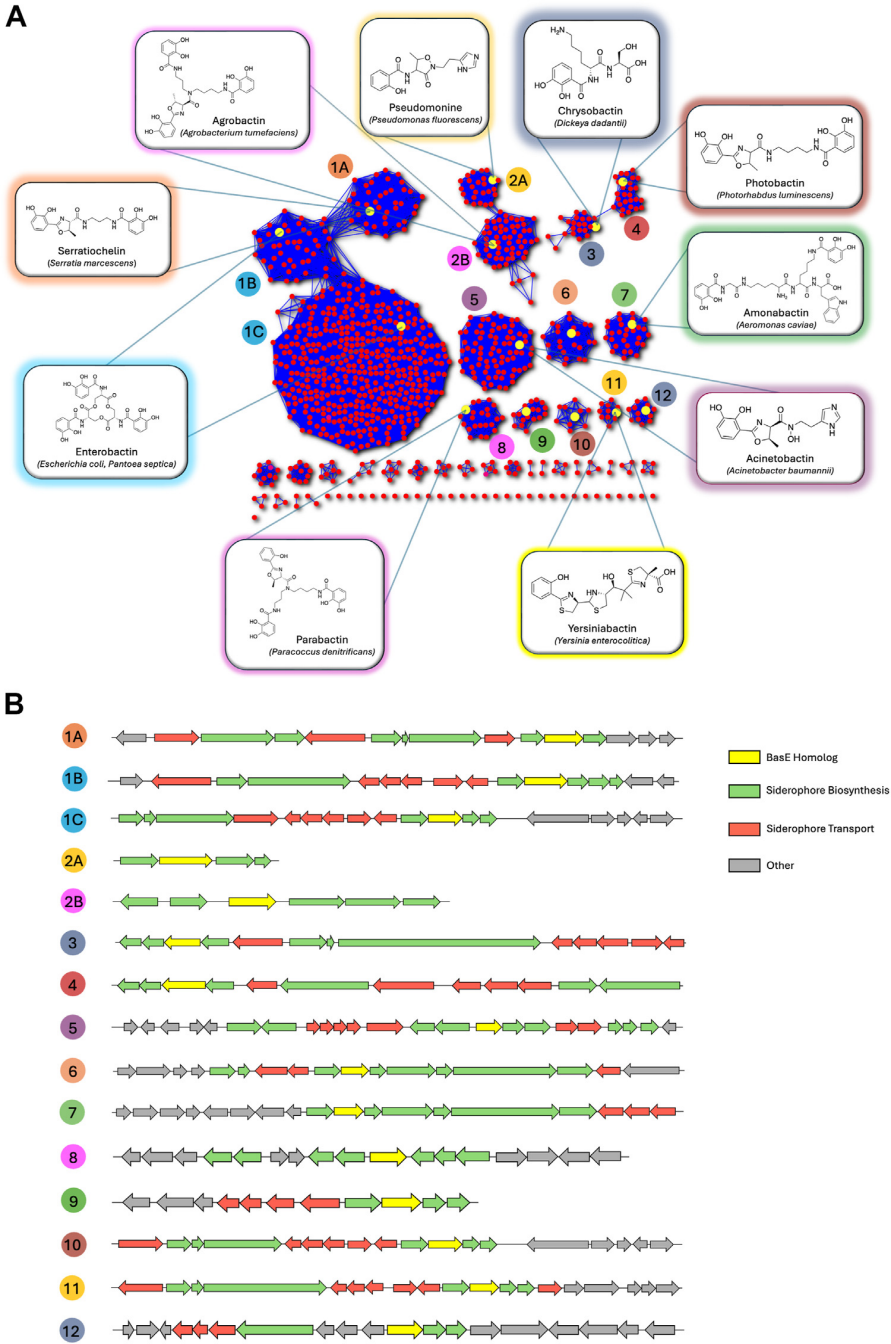
**Sequence similarity network of BasE homologs from Gram-negative bacteria.***A*, the top 1000 BasE homologs with each node representing a unique protein and each edge representing 75% sequence similarity. Each cluster produces structurally unique catecholate or phenolate siderophores. *B*, representative genome neighborhood diagrams for the 12 largest clusters depict diversity in genome architecture of different siderophore biosynthetic pathways.

Motivated to identify functionally characterized homologs of BasE as well as homologous NRPS pathways, we generated a sequence similarity network (SSN) using Enzyme Function Initiative-Enzyme Similarity Tool (EFI-EST) (Fig. 6*A*) (60). The initial EFI-BLAST search was limited to bacterial species with an alignment score of 100. The top 1000 homologs of BasE were categorized based on sequence similarity with a grouping cutoff of 75% pairwise sequence identity when visualizing the network edges, resulting in 12 major clusters as well as several minor clusters. The SSN allowed us to identify several homologous standalone adenylation domains, each playing roles in unique catecholate siderophore biosynthetic pathways. These siderophores are structurally diverse and have broad distribution across bacterial taxonomies. We also used the EFI-genome neighborhood tool (61) to visualize the genome architecture of each identified siderophore pathway (Fig. 6*B* and Table S1). Our analysis also identified several structurally unique siderophores, the pathways of which have not been well characterized. Examples of these are parabactin—which has both catecholate and phenolate moieties, and pseudomonine—which is structurally similar to acinetobactin but is capped by a phenolate moiety. Studying these homologous siderophores could open new avenues for NRPS engineering to generate enzymes that can recognize both non-native substrates as well interact with non-native biosynthetic pathways. In addition, several of the identified siderophores are produced by AMR Gram-negative bacteria, suggesting that these could potentially be targets for the synthesis of novel antimicrobials in the future.

Our study establishes a foundation for creating novel acinetobactin derivatives with antimicrobial properties, offering new possibilities for siderophore-based antimicrobials against antibiotic-resistant bacteria like *A. baumannii*. By testing these derivatives for antimicrobial activity or as building blocks for conjugation with existing antibiotics, the potential for novel, more effective treatments is expanded. Furthermore, the application of chemoenzymatic methods, where enzymes replace complex chemical synthesis steps, further strengthens this approach. Enzymes provide a precise, efficient means to modify complex molecules under mild conditions, which is not only more cost effective but also eco-friendly, reducing reliance on toxic chemicals and minimizing waste. This shift to enzyme-based synthesis could significantly streamline the discovery of natural product analogs, allowing for faster, scalable production of novel natural product derivatives. The use of a relatively small NRPS system for a siderophore will, we hope, establish the foundation for future studies that advance this approach to more complex NRPS systems.

Our findings emphasize that enzyme engineering will be critical to maximizing the potential of these chemoenzymatic strategies. By tailoring enzyme functions to generate specific modifications, we can expand the range of natural product analogs available, fine-tuning their structures for increased effectiveness against resistant strains. Enzyme centered approaches such as this could transform drug discovery pipelines, providing a versatile platform for developing targeted antimicrobials to combat multidrug-resistant bacteria effectively and sustainably.

## Experimental procedures

### Cloning and gene annotation

Cloned WT *basE* from clinical strain *A. baumannii* AB900 in pET15bTEV vector containing the His-tag was obtained for heterologous expression in *Escherichia coli* (GenBank Accession: WP_000744385.1). Cloning and sequence analysis of *basE* was conducted in previous studies, which included the introduction of a point mutation P45L that lies on the surface of the protein, 25 Å from the active site (55). BasE variants for the current study were generated using site-directed mutagenesis using PCR. Oligonucleotides used for mutagenesis are shown in Table S2.

### Large-scale protein expression and purification

WT BasE in pET-15bTEV vector (Tables S3 and S4) was transformed into *E. coli* BL21 DE3 cells. Cells were grown overnight in 50 ml LB containing 50 μg/ml ampicillin at 37 °C. Ten microliters of overnight culture was then inoculated into 1 L LB medium supplemented with 50 μg/ml ampicillin. Cultures were grown at 37 °C with agitation until the A_600_ reached 0.6 to 0.8 when they were cooled at 4 °C for 10 min. Protein expression was induced with 0.5 mM IPTG and cultures were grown for 18 h at 20 °C. Cells were cooled at 4 °C and harvested *via* centrifugation at 6000 rpm for 10 min and frozen at −80 °C. The following purification steps were carried out at 4 °C.

His-tagged BasE was purified using immobilized metal affinity chromatography. Cells were resuspended in lysis buffer (50 mM Hepes pH 7.5, 500 mM NaCl, 0.2 mM TCEP, 20 mM imidazole, and 10% glycerol) and lysed using sonication (5 s ON, 10 s OFF for a total on time of 5 min at 60% amplitude). The resulting cell lysate was centrifuged at 40,000 rpm for 40 min. The supernatant was decanted and filtered using a 0.45 μm filter. The filtrate was passed over a 5 ml nickel-loaded HisTrap column. The column was washed with 10 column volumes of lysis buffer followed by 5 column volumes of 6% elution buffer (50 mM Hepes pH 7.5, 500 mM NaCl, 0.2 mM TCEP, 300 mM imidazole, and 10% glycerol) to remove loosely bound contaminants. The immobilized protein was then eluted with 15 column volumes of elution buffer (gradient) and dialyzed overnight with dialysis buffer (20 mM Hepes pH 7.5, 100 mM NaCl, and 0.2 mM TCEP). The dialyzed protein was collected the next day and concentrated using a 15 ml Amicon Centrifugation filter (30 kDa molecular weight cutoff) until the concentration reached 10 to 20 mg/ml. The concentrated protein was spun down at 14,000 rpm to remove the presence of any aggregates. The protein was then flash-frozen in liquid nitrogen and stored at −80 °C. The same protocol was used for the BasE mutants.

### Crystallization and structure solution of BasE mutants

His-tagged BasE purified *via* nickel affinity chromatography was used for crystallographic trials. A sparse matrix crystal screen was created using a high-throughput Crystal Gryphon robot with 10 mg/ml and 20 mg/ml BasE in the presence of 2 to 5 mM substrates at 14 °C. Initial crystal hits of 20 mg/ml BasE were observed within a week of setting up trays, in the presence of 25% PEG 4000, 0.2 M calcium chloride, and 0.1 M Tris–HCl pH 8.5. After optimization, crystals were observed at 12% PEG 4000, 0.1 M calcium chloride, and 0.05 M Tris–HCl pH 8.5. Identical conditions were used for cocrystallization with 3 mM 4-methylsalicylic acid, 3 mM 4-fluorosalicylic acid, and 5 mM 4-azidosalicylic acid. Crystals were harvested within a week of observation and cryoprotected with crystallization cocktail containing ligands and 16% ethylene glycol and flash-frozen in liquid nitrogen.

Diffraction data for BasE single crystals were collected remotely at SSRL using beamline BL12-2. Datasets were processed at 2.0 to 2.5 Å using Xia2/Dials in the P2_1_2_1_2_1_ space group. Structures were solved *via* molecular replacement with Phaser in the PHENIX Suite using a single chain of the previously solved WT structure of BasE (55) as the search model (PDB 3O83), with the side chains of mutated residues truncated and all solvent and ligand atoms removed. Because the C-terminal subdomain was disordered in the WT structure, the search model consisted of only the N-terminal subdomain (residues 1–439). This resulted in solutions containing two molecules per asymmetric unit. No extra density was observed for the dynamic C-terminal subdomain of each monomer, which was not included in the final model. Manual model building and refinement were carried out using Phenix.refine (62) and COOT (63). The volume of BasE binding pockets was analyzed using HOLLOW (64). Molecular docking was conducted using AutoDock Vina (65) within the UCSF Chimera suite (66). Diffraction and refinement data are presented in Table S5.

### Analysis of BasE adenylation activity *via* the NADH consumption assay

The adenylation activity of the WT and mutant BasE enzymes was measured using the commonly used adenylation assay (67) that couples AMP formation to NADH oxidation through the use of coupling enzymes myokinase, pyruvate kinase, and lactate dehydrogenase (Fig. S1). To avoid having to use the holo-carrier protein domain of BasF as the acceptor substrate, we used hydroxylamine, which is commonly used as a surrogate substrate in adenylate-forming enzymes (68) including NRPS adenylation domains (38, 69, 70, 71), to convert the adenylate intermediate to AMP. The reaction mixture contained 100 mM Hepes pH 7.5, 15 mM MgCl_2_, 3 mM ATP, 50 mM hydroxylamine, 3 mM phosphoenolpyruvate, 0.8 mM NADH, and 10 units/ml of each coupling enzyme myokinase, pyruvate kinase, lactate dehydrogenase, and varying concentrations of substrates and BasE WT and variants. For the initial specificity screening, 1 mM of each substrate and 3 μM enzyme was used. However, for steady-state kinetic measurements, the substrate and enzyme concentration varied and was typically 0.2 to 5 μM for enzyme with 6 to 8 different substrate concentrations (per assay). The assay was conducted in 100 μl reaction volume in clear 96-well polystyrene plates and all reactions were done in triplicates. The master mix was first incubated for 10 min at 37 °C without the presence of protein or substrate. In a separate plate, 10 μl of protein and substrate were added making sure they did not contact each other. Once the master mix was incubated, 80 μl was added to wells containing protein and substrate. NADH absorbance was continuously monitored at 340 nm for 20 min at 37 °C using a BioTek Cytation 1 plate reader. These absorbance values were converted to concentrations of NADH using the molar extinction coefficient of NADH (6220 M^-1^ cm^-1^). Using GraphPad Prism 10 (https://www.graphpad.com), the rate of decay of NADH was used to generate specific activity plots for substrate screening. The slopes of these specific activity plots for different substrate concentrations were then used to generate Michaelis–Menten plots using the nonlinear regression model for Michaelis–Menten kinetics which is V = Vmax[S]/(K_M_ + [S]). We note that with poor substrates, the maximum reaction rate may be influenced by the ability of the adenylate intermediate to leak off the enzyme, where it can react with hydroxylamine in solution (70).

### Sequence similarity network

An SSN for aryl adenylating enzymes was generated using EFI-EST using the BasE sequence with a query e-value of 5 (60). The top 1000 sequences retrieved using the BLAST mode of EFI-EST were used to generate an SSN using an alignment score of 100. The resulting 100% representative network was obtained and visualized using Cytoscape (72). A filter of 75% identity was employed to reduce the number of edges in the network. The final SSN consisted of 1000 nodes each representing a unique sequence, from which 13 nodes were further annotated. The EFI-genome neighborhood tool (60) was used to create the genome neighborhood diagrams.

## Data availability

The structures of BasE have been deposited with the worldwide Protein Data Bank: BasE V336A mutant bound to 4-methylsalicylic acid (9MY5), BasE V336G mutant bound to 4-aminosalicylic acid (9MY7), and BasE S247C/V336A mutant bound to 4-fluorosalicylic acid (9MY6).

## Supporting information

This article contains supporting information, including Michaelis–Menton plots, docking, and structural alignments, diffraction and structural refinement tables, and gene and protein sequence information.

## Conflict of interest

A. M. G. is an Editorial Board Member for this journal. He was not involved in the editorial review or the decision to publish this article. The other author declares that he has no conflicts of interest with the contents of this article.
