# Supplementary material for: The structural basis of substrate selectivity of the acinetobactin biosynthetic adenylation domain, BasE
Source: J Biol Chem. 2025 Mar 15;301(4):108413. doi: 10.1016/j.jbc.2025.108413 (PMC12005286; doi:10.1016/j.jbc.2025.108413)
Supplement: Supplemental Material [file mmc1.pdf]

The Structural Basis of Substrate Selectivity of the Acinetobactin Biosynthetic Adenylation Domain, BasE

Supplemental Information

Syed Fardin Ahmed and Andrew M. Gulick

Department of Structural Biology, University at Buffalo, Buffalo, NY, 14203, United States.

Table of Contents.

Supplemental Figures **2**

Supplemental Figure S1. Adenylation domain biochemical assay 2

Supplemental Figure S2. Michaelis-Menten Kinetics of wildtype BasE 3

Supplemental Figure S3. Michaelis-Menten Kinetics of S247C mutant of BasE 4

Supplemental Figure S4. Michaelis-Menten Kinetics of V336A mutant of BasE 5

Supplemental Figure S5. Michaelis-Menten Kinetics of V336G mutant of BasE 6

Supplemental Figure S6. Michaelis-Menten Kinetics of Y346A mutant of BasE 7

Supplemental Figure S7. Michaelis-Menten Kinetics of V336A/Y346A mutant of BasE 8

Supplemental Figure S8. Michaelis-Menten Kinetics of V336A/S247C mutant of BasE 9

Supplemental Figure S9. Molecular docking of 4-azidosalicylic acid to BasE V336G. 10

Supplemental Figure S10. Alignment of crystal structures of BasE mutants 11

Supplemental tables ***12***

Supplemental Table S1. Specificity codes residues of adenylation domains across different families of gram-negative bacteria identified from sequence similarity network (SSN) 12

Supplemental Table S2. Primers used in this study 13

Supplemental Table S3. Gene encoding BasE 14

Supplemental Table S4. Protein sequence for BasE construct from strain AB900. 15

Supplemental Table S5. Crystallographic data collection and refinement statistics 16

Supplemental Figures

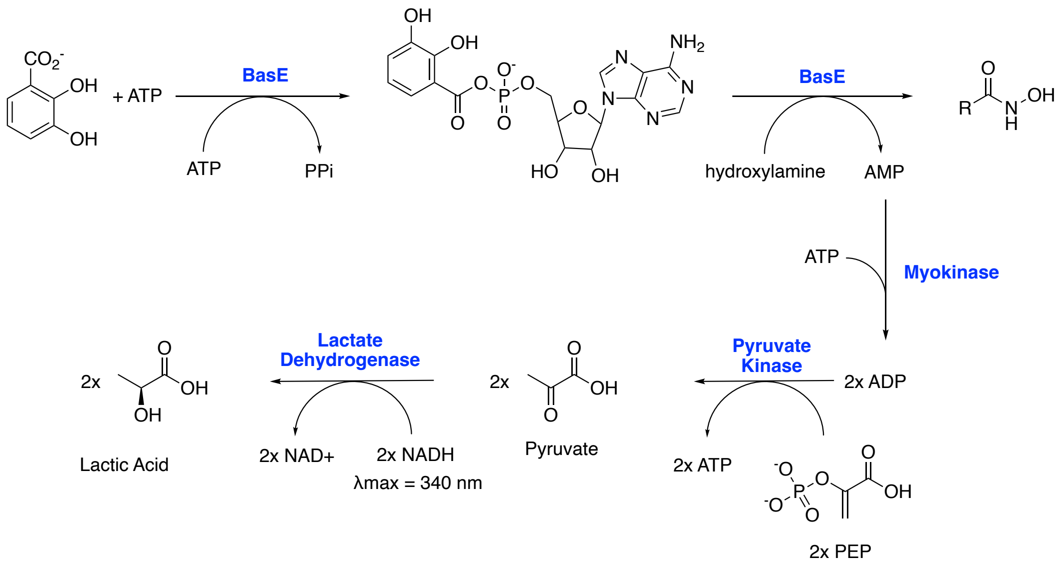

Supplemental Figure S1. Adenylation domain biochemical assay. All kinetic data were collected using the NADH consumption assay, a coupled enzymatic assay to measure adenylation rates through measuring AMP release. BasE converts the aryl acid into an adenylated intermediate using ATP to form an aryl adenylate and PPi. In the natural reaction, the pantetheine cofactor of the carrier domain of BasF attacks the adenylate intermediate to load the substrate and release AMP. Here, hydroxylamine is used as a surrogate nucleophile to form an aryl hydroxamate along with AMP. The rate of AMP formation is coupled to $NADH^+$ consumption through the activities of myokinase, pyruvate kinase, and lactate dehydrogenase. Myokinase converts the AMP and ATP to two ADP molecules. Each ADP molecule is used by pyruvate kinase to convert phosphoenolpyruvate (PEP) to pyruvate, which is then converted by lactate dehydrogenase to lactate, with associated oxidation of NADH to NAD^+. The oxidation of NADH to NAD^+ is monitored at 340 nm and converted to initial velocity values in µM/min using $\varepsilon_{340} = 6220\ M^{-1}\ cm^{-1}$.

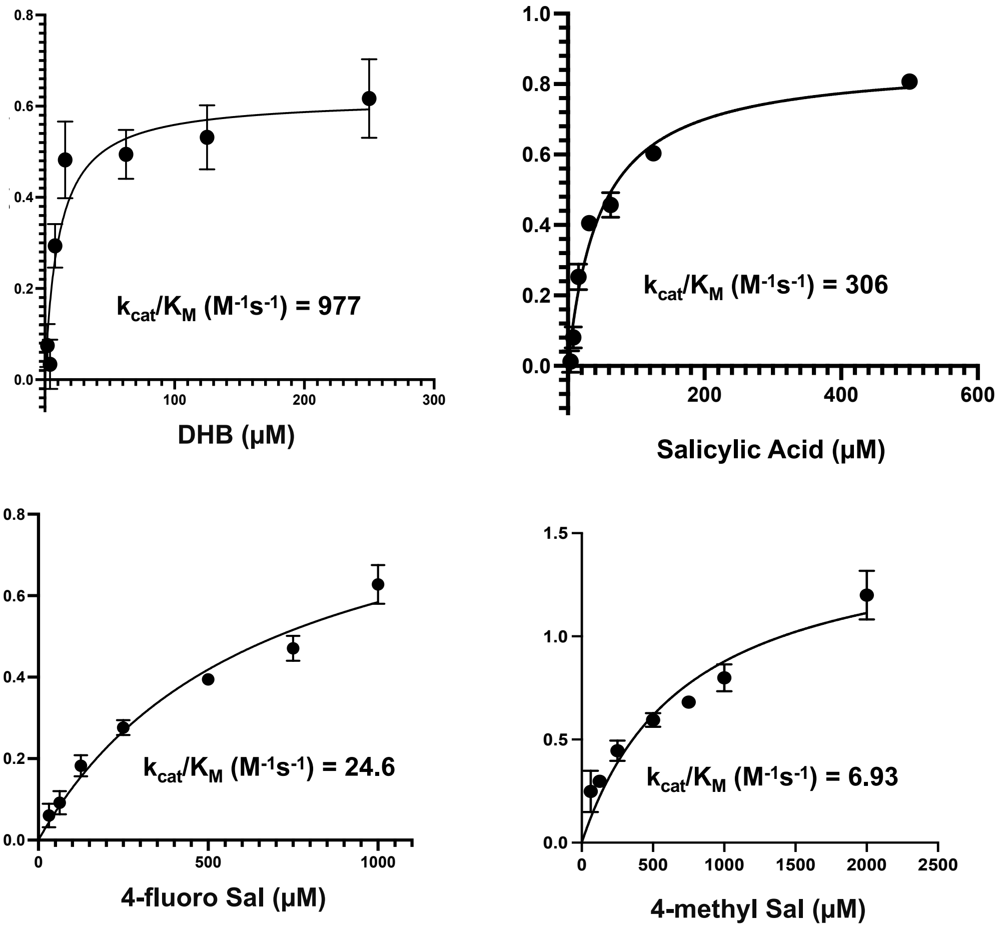

Supplemental Figure S2. Michaelis-Menten Kinetics of wildtype BasE. Initial velocity plots were created for wildtype BasE against a variety of substrate analogs. Data points represent three replicates performed with 1 µM enzyme concentration. For 4-methylsalicylic acid, 5 µM enzyme was used.

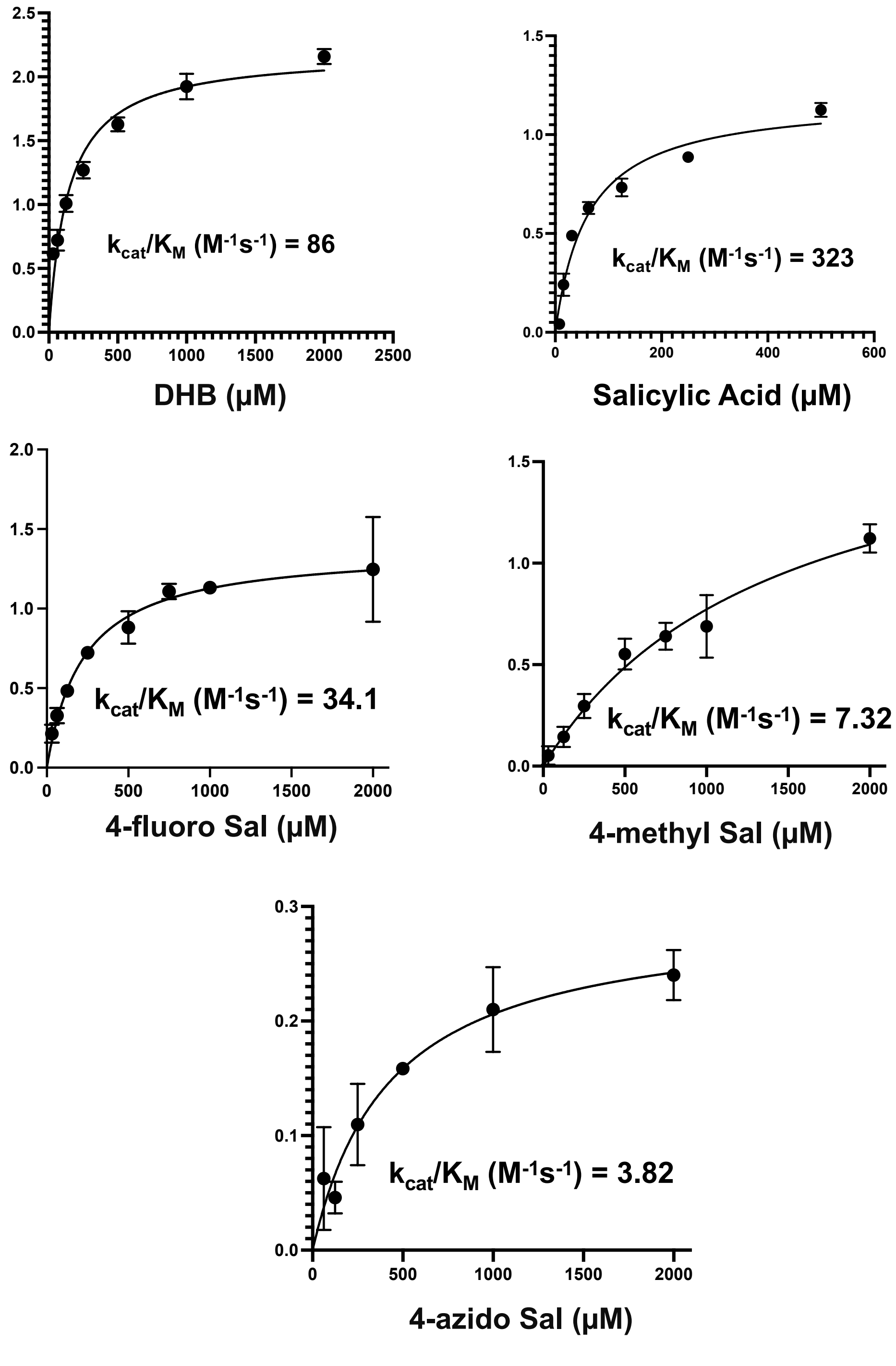

Supplemental Figure S3. Michaelis-Menten Kinetics of S247C mutant of BasE. Initial velocity plots were created for FbsH S247C against a variety of substrate analogs. Data points represent three replicates performed with 3 µM enzyme concentration. For salicylic acid, 1 µM enzyme was used.

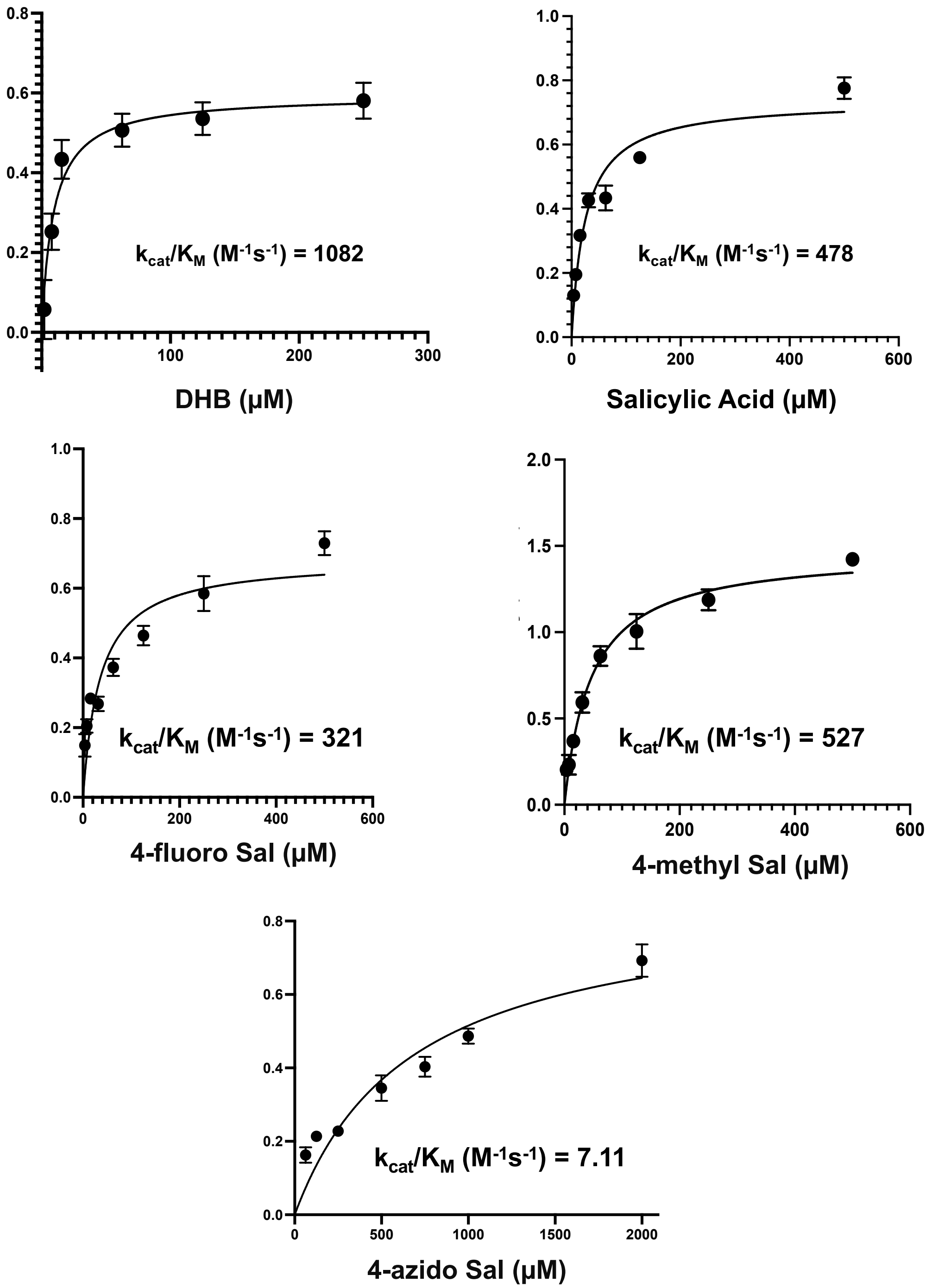

Supplemental Figure S4. Michaelis-Menten Kinetics of V336A mutant of BasE. Initial velocity plots were created for FbsH V336A against a variety of substrate analogs. Data points represent three replicates performed with 1 µM enzyme concentration. For 4-azidosalicylic acid, 3 µM enzyme was used.

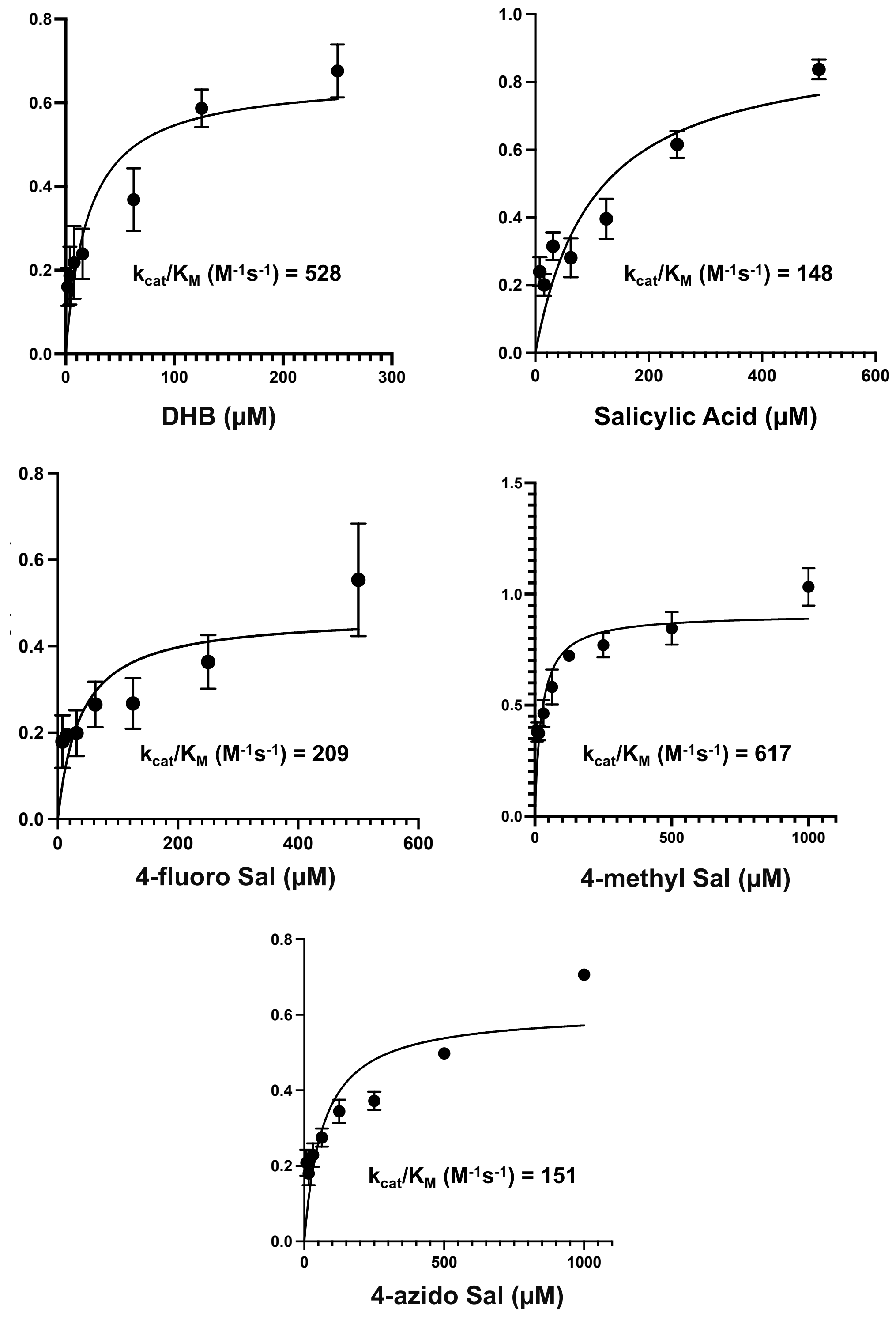

Supplemental Figure S5. Michaelis-Menten Kinetics of V336G mutant of BasE. Initial velocity plots were created for FbsH V336G against a variety of substrate analogs. Data points represent three replicates performed with 1 µM enzyme concentration.

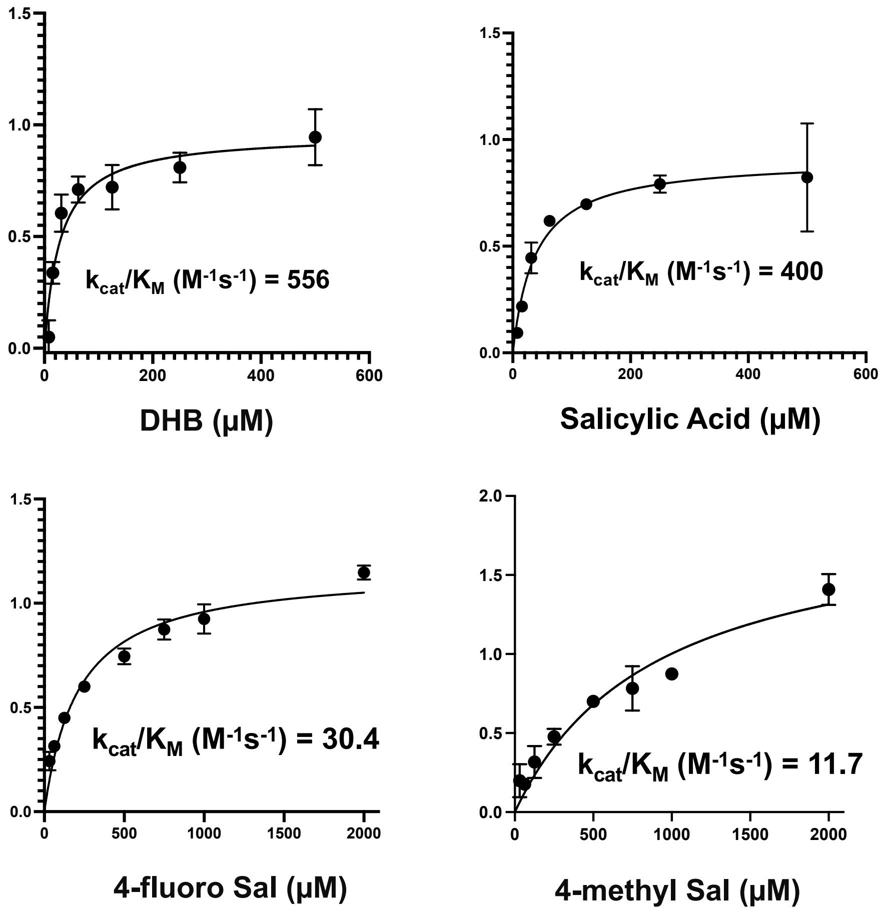

Supplemental Figure S6. Michaelis-Menten Kinetics of Y346A mutant of BasE. Initial velocity plots were created for FbsH Y346A against a variety of substrate analogs. Data points represent three replicates performed with 1 µM enzyme concentration. For 4-methylsalicylic acid and 4-fluorosalicylic acid, 3 µM enzyme was used.

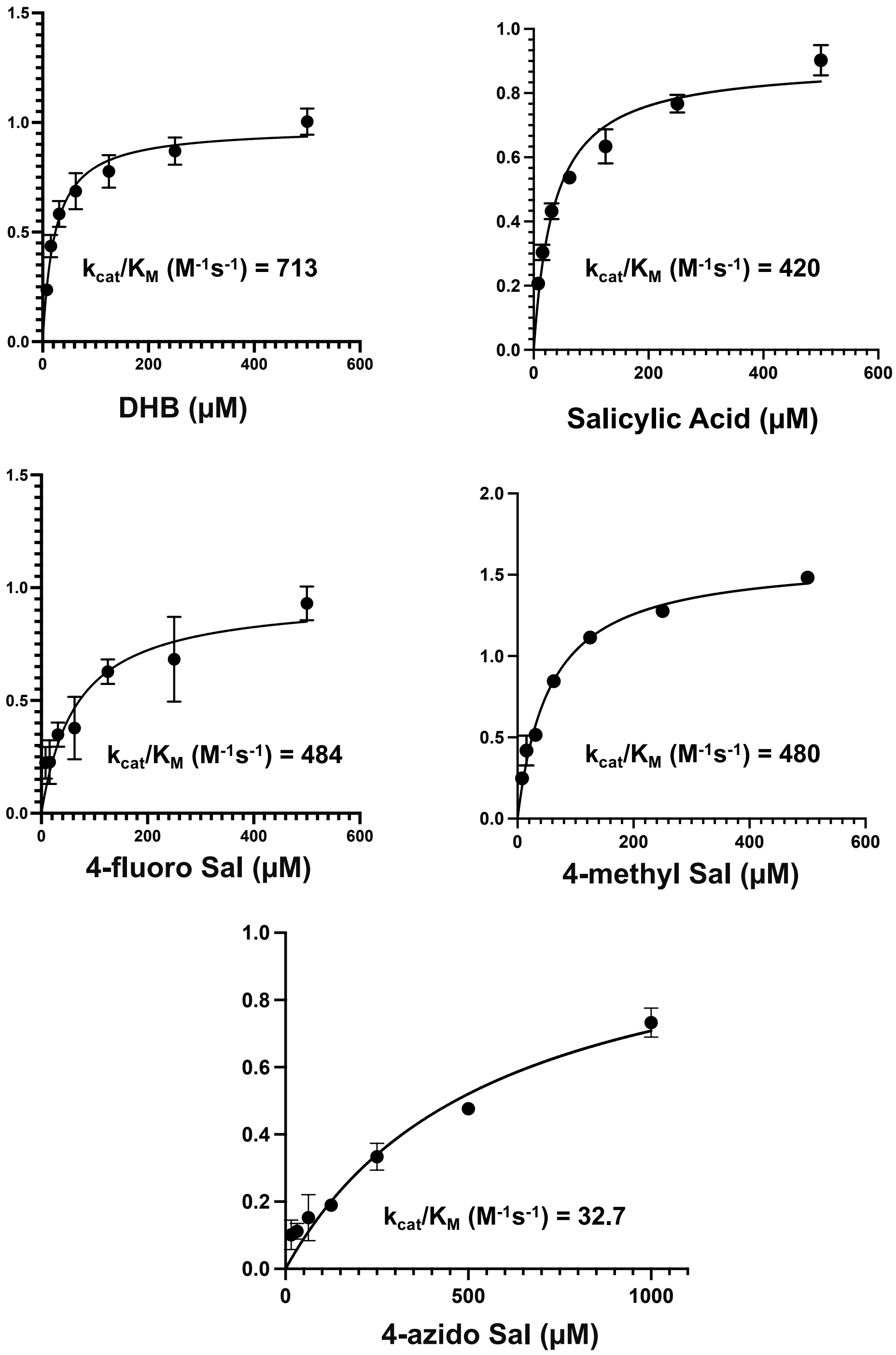

Supplemental Figure S7. Michaelis-Menten Kinetics of V336A/Y346A mutant of BasE. Initial velocity plots were created for FbsH V336A/Y346A against a variety of substrate analogs. Data points represent three replicates performed with 1 μM enzyme concentration. For 4-fluorosalicylic, 0.5 μM enzyme was used.

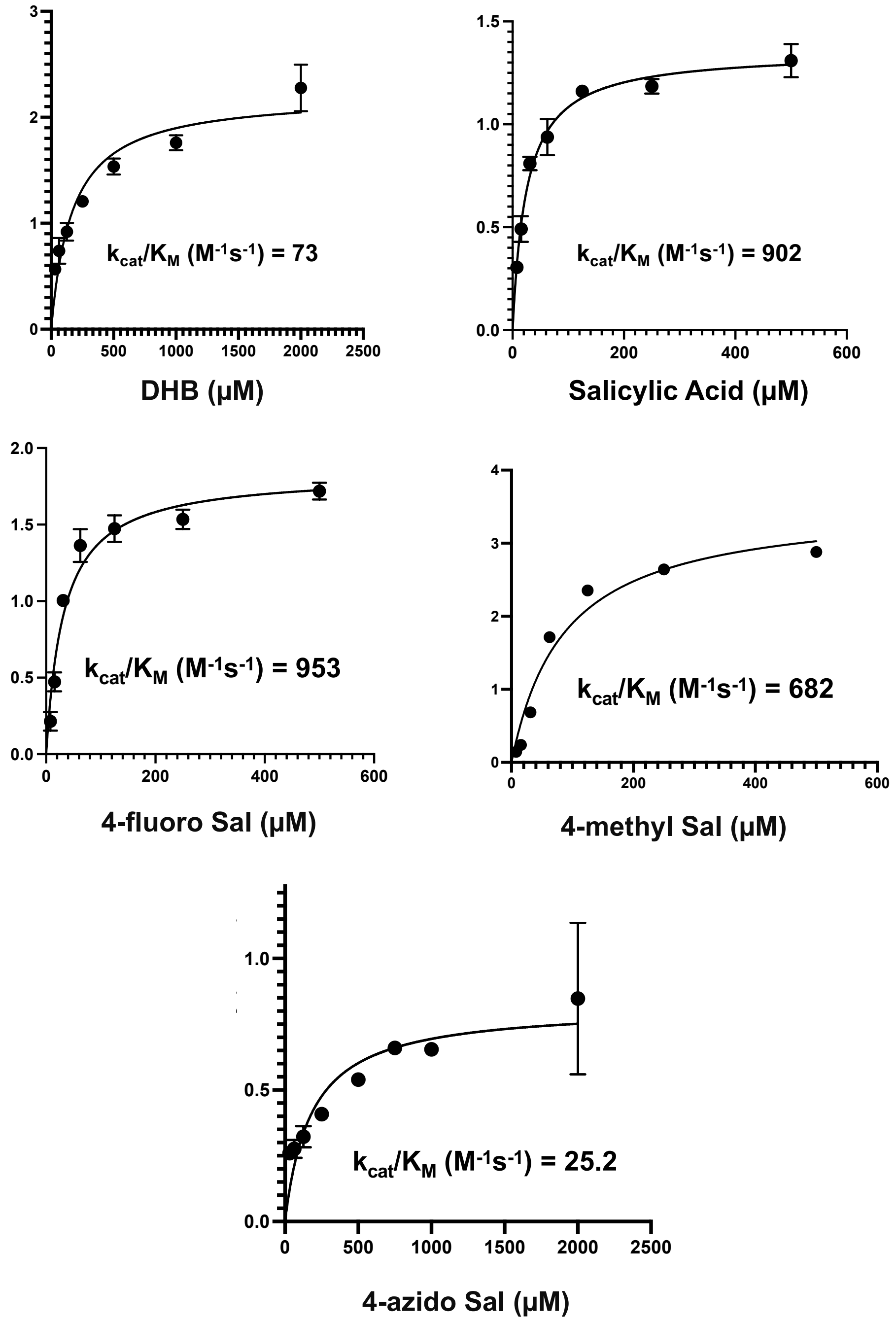

Supplemental Figure S8. Michaelis-Menten Kinetics of V336A/S247C mutant of BasE. Initial velocity plots were created for FbsH V336A/S247C against a variety of substrate analogs. Data points represent three replicates performed with 1 μM enzyme concentration. For DHB and 4-azidosalicylic acid, 5 μM enzyme was used.

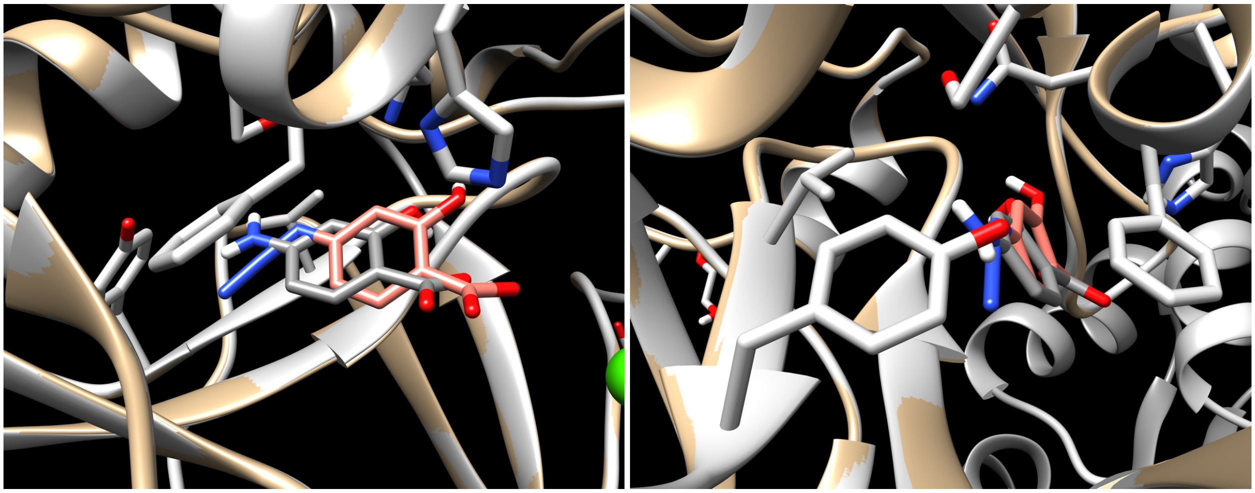

Supplemental Figure S9. Molecular docking of 4-azidosalicylic acid to BasE V336G.
The conformation of 4-aminosalicylic acid from crystal structure (grey) shows similarity with docked ligand (red).

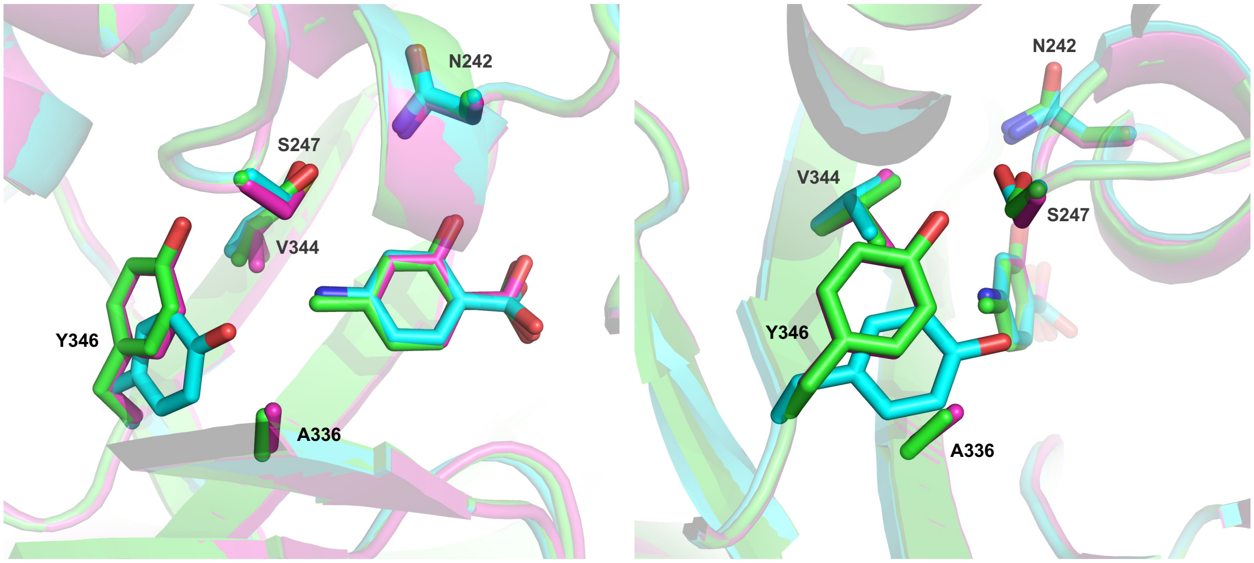

Supplemental Figure S10. Alignment of crystal structures of BasE mutants.
The three mutants are V336A bound to 4-methylsalicylic (green), V336G bound to 4-aminosalicylic acid (cyan) and S247C/V336A bound to 4-fluorosalicylic acid (pink).

Supplemental tables

Supplemental Table S1. Specificity codes residues of adenylation domains across different families of gram-negative bacteria identified from sequence similarity network (SSN)

Cluster	Representative Organism	Siderophore	Family	Specificity Code
1A	*Serratia marcescens*	Serratiochelin	Yersiniaceae	RVLGRSNVLK
1B	*Escherichia coli*	Enterobactin	Enterobacteriaceae	RVLGRSNALK
1C	*Pantoea septica*	Enterobactin	Erwiniaceae	RVLGRSNALK
2A	*Pseudomonas fluorescens*	Pseudomonine	Pseudomonadaceae	DILGRSNALK
2B	*Agrobacterium tumefaciens*	Agrobactin	Rhizobiaceae	AVLGRSNALK
3	*Dickeya dadantii*	Chrysobactin	Pectobacteriaceae	QQLGRSNALK
4	*Photorhabdus luminescens*	Photobactin	Morganellaceae	SHLGRSNALK
5	*Acinetobacter baumannii*	Acinetobactin	Moraxellaceae	EIMGRSNALK
7	*Aeromonas caviae*	Amonabactin	Aeromonadaceae	ELLGRSNALK
8	*Paracoccus denitrificans*	Parabactin	Paracoccaceae	AHLGRSNALK
11	*Yersinia enterocolitica*	Yersiniabactin	Yersiniaceae	EFMGRSNALK

Supplemental Table S2. Primers used in this study

Primer name	Sequence 5'-3'
BasE_V344I_F	CCGAAGGCTTGATCAATTACACCAGACTCGATGAC
BasE_V344I_R	GTAATTGATCAAGCCTTCGGCCATTCCAAATACTTG
BasE_V344L_F	CCGAAGGCTTGCTCAATTACACCAGACTCGATGAC
BasE_V344L_R	GTAATTGAGCAAGCCTTCGGCCATTCCAAATACTTG
BasE_Y346A_F	GCTTGGTCAATGCCACCAGACTCGATGACTCCGACGAG
BasE_Y346A_R	GTCTGGTGGCATTGACCAAGCCTTCGGCCATTCCAAATAC
BasE_S247C_F	GTTGAGTTGCCCCGGTGCATTAGGCGTTTTAC
BasE_S247C_R	CACCGGGGCAACTCAACATAAAGTTATGCGGAG
BasE_F243L_F	CCGCATAACCTTATGTTGAGTTCACCCGGTGC
BasE_F243L_R	CTCAACATAAGGTTATGCGGAGCTGGCAGAGCGCAC
BasE_V336A_F	TACAGCAGGCATTTGGGATGGCCGAAGGCTTGGTC
BasE_V336A_R	CCCAAATGCCTGCTGTAATTTACAATTGAGAACTTC
BasE_V336G_F	TACAGCAGGGATTTGGGATGGCCGAAGGCTTGGTC
BasE_V336G_R	CCCAAATCCCTGCTGTAATTTACAATTGAGAACTTC
BasE_Y346A/V336A_F	TACAGCAGGCATTTGGGATGGCCGAAGGCTTGGTC
BasE_Y346A/V336A_R	CCCAAATGCCTGCTGTAATTTACAATTGAGAACTTC
BasE_S247C/V336A_F	TACAGCAGGCATTTGGGATGGCCGAAGGCTTGGTC
BasE_S247CV336A_R	CCCAAATGCCTGCTGTAATTTACAATTGAGAACTTC

Supplemental Table S3. Gene encoding BasE

Gene	Sequence
basE<br><br>*Protein Entry*<br>WP_000744385.1<br><br>*Nucleotide Entry*<br>NZ_JAAVKC010000001.1<br>(239933 – 241561)	ATGAAAAAACAGTTGATTGAGTTTGTTCGCTGGTCTCCAGAGAGAGCGCAACACTATCG<br>AAATAAAGGTTATTGGATTGACCAACCTTTGACGCGCATTTTAACGGTAGGAGTTCAAT<br>CGCATCCGCATTCGCTGGCAATTATTTGTGGTGAGCGTCAGTTGAGTTATATCGAGCTG<br>GACCGGCTATCAACAAATTTAGCTACTCGACTAGCTGAAAAAGGCTTGGGCAAGGGCGA<br>TACTGCTTTAGTACAACTTCCCAATGTTGCAGAGTTTTATATTGTCTTTTTTGCCTTGC<br>TTAAAGCAGGCGTGGTTGTGCTCAATGCGCTATATAGCCATCGCCAATATGAACTAAAT<br>GCCTTTATTAAACAGATTCAACCTAAGCTTTTAATTGGTTCGCGTCAGCATGAAGTATT<br>TAGCAATAATCAATTTATTGATTCACTTCATGATGTAAATTTAAGTCCTGAAATTATTT<br>TGATGCTCAATCATCAAGCTACCGATTTCGGACTATTAGACTGGATTGAAACACCAGCA<br>GAGACCTTTGTCGATTTTTCATCTACACCTGCTGATGAAGTTGCTTTCTTTCAGTTGTC<br>AGGTGGAAGTACGGGAACACCAAAACTTATTCCACGCACGCATAATGACTATGACTATA<br>GTGTGCGAGCCAGTGCTGAGATTTGCGGTTTAAACTCAAATACCCGGTTATTGTGCGCT<br>CTGCCAGCTCCGCATAACTTTATGTTGAGTTCACCCGGTGCATTAGGCGTTTTACATGC<br>AGGTGGATGTGTTGTGATGGCACCAAATCCTGAACCGCTCAATTGTTTTTCTATTATTC<br>AAAGACATCAAGTCAATATGGCATCTTTAGTGCCGAGTGCTGTCATTATGTGGCTAGAA<br>AAAGCCGCGCAATATAAAGATCAAATTCAGTCTTTAAAGTTGCTTCAAGTAGGTGGAGC<br>AAGTTTCCCTGAATCTTTAGCCCGTCAAGTTCCTGAAGTTCTCAATTGTAAATTACAAC<br>AAGTATTTGGAATGGCCGAAGGCTTGGTCAATTACACCAGACTCGATGACTCCGACGAG<br>CAAATCTTTACGACACAAGGGCGTCCTATCAGTTCTGATGATGAAATCAAAATTGTGGA<br>TGAACAGTATAGAGAGGTTCCAGAAGGTGAAATAGGGATGCTTGCGACTCGGGGACCTT<br>ATACCTTCTGTGGTTATTACCAAAGCCCTGAACATAATTCACAGGTCTTTGATGAGGAC<br>AACTATTACTATTCGGGCGATCTCGTGCAGCGTACCCCTGATGGTAATTTACGTGTAGT<br>AGGAAGAATTAAAGACCAGATTAACCGTGGTGGTGAAAAGATTGCTTCGGAAGAAATAG<br>AAAAACTTATTCTTCTACATCCGGAAGTTATGCACGCAGCTTTGGTCGCAATTGTTGAT<br>GAACAATTTGGTGAAAAAAGTTGTGCCTTTATTGTTTCTCGTAATCCTGAACTTAAAGC<br>TGTTGTGCTCAGACGCCATCTTATGGAGTTGGGTATTGCACAATACAAACTTCCAGACC<br>AGATCAAATTAATCGAAAGTTTGCCACTGACTGCAGTCGGTAAGGTGGACAAAAAACAA<br>CTTCGCAGCATTTTAAATACATCTACAACATCTTAA

Supplemental Table S4. Protein sequence for BasE construct from strain AB900.

Protein	Sequence
BasE<br>WP_000744385.1	MGSSHHHHHHSSGENLYFQGHMKKQLIEFVRWSPERAQHYRNKGYWIDQPLTRILTVGVQ<br>SHPHSLAIICGERQLSYIELDRLSTNLATRLAEKGLGKGDTALVQLPNVAEFYIVFFAL<br>LKAGVVVLNALYSHRQYELNAFIKQIQPKLLIGSRQHEVFSNNQFIDSLHDVNLSPEII<br>LMLNHQATDFGLLDWIETPAETFVDFSSTPADEVAFFQLSGGSTGTPKLIPRTHNDYDY<br>SVRASAEICGLNSNTRLLCALPAPHNFMLSSPGALGVLHAGGCVVMAPNPEPLNCFSII<br>QRHQVNMASLVPSAVIMWLEKAAQYKDQIQSLKLLQVGGASFPESLARQVPEVLNCKLQ<br>QVFGMAEGLVNYTRLDDSDEQIFTTQGRPISSDDEIKIVDEQYREVPEGEIGMLATRGP<br>YTFCGYYQSPEHNSQVFDEDNYYYSGDLVQRTPDGNLRVVGRIKDQINRGGEKIASEEI<br>EKLILLHPEVMHAALVAIVDEQFGEKSCAFIVSRNPELKAVVLRRHLMELGIAQYKLPD<br>QIKLIESLPLTAVGKVDKKQLRSILNTSTTS

Sequence highlighted yellow represents N-6x-His-tag and TEV cleavage site residues (ENLYFQ^G) contributed by vector pET15b_TEV.

Supplemental Table S5. Crystallographic data collection and refinement statistics

DATA COLLECTION	V336A, 4-methyl Sal	V336G, 4-amino Sal	S247C/V336A, 4-fluoro Sal
PDB CODE	**9MY5**	**9MY7**	**9MY6**
Beamline	SSRL BL12-2	SSRL BL12-2	SSRL BL12-2
Wavelength (Å)	0.97946	0.97946	0.97946
Resolution range (Å)	39.59 - 2.39 (2.46 - 2.39)	66.17 - 2.53 (2.67 - 2.53)	102.33 - 2.07 (2.19 - 2.07)
Space group	$P2_12_12_1$	$P2_12_12_1$	$P2_12_12_1$
a, b, c (Å)	65.76 143.68 148.72	65.29 143.35 149.20	65.42 141.25 148.46
α, β, γ (°)	90.00 90.00 90.00	90.00 90.00 90.00	90.00 90.00 90.00
Total reflections	389436 (32409)	185503 (27356)	384818 (59861)
Unique reflections	56567 (4575)	47313 (6822)	80620 (12154)
Multiplicity	6.9 (7.1)	3.9 (4.0)	4.8 (4.9)
Completeness (%)	99.8 (100.0)	99.6 (99.9)	95.8 (99.8)
Mean I/sigma(I)	14.0 (1.6)	9.6 (2.9)	10.8 (2.1)
R_{merge}	0.093 (1.462)	0.113 (1.869)	0.079 (1.068)
R_{pim}	0.041 (0.629)	0.063 (1.008)	0.040 (0.534)
$CC_{1/2}$	0.999 (0.728)	0.996 (0.522)	0.995 (0.853)
**Refinement**			
Resolution range (Å)	39.59 - 2.39 (2.46 - 2.39)	66.17 - 2.53 (2.67 - 2.53)	63.78 - 2.073 (2.12 - 2.07)
Reflections, refinement	56423 (3968)	47067 (3224)	80151 (5872)
Reflections, R_{free}	2001 (148)	1996 (130)	1997 (147)
R_{work}	0.1862 (0.3052)	0.2132 (0.3699)	0.1919 (0.3132)
R_{free}	0.2254 (0.3499)	0.2631 (0.4298)	0.2184 (0.3515)
Protein residues	872	870	872
Ligands of interest	2 A1BUB	2 BHA	2 OOI
Other molecules	6 EDO, 7 Ca^{2+}	4 EDO, 9 Ca^{2+}	1 EDO, 2 PEG
Water molecules	288	153	214
Rms (bonds) (Å)	0.009	0.006	0.011
Rms (angles) (°)	0.92	0.50	1.08
Rama. Favored (%)	97.47	96.54	98.04
Rama. Allowed (%)	2.42	3.46	1.96
Rama. Outliers (%)	0.12	0.00	0.00
Rotamer outliers (%)	1.82	1.01	0.83
Average B-factor ($Å^2$)	58.06	70.40	52.37
macromolecules	58.08	70.49	52.47
ligands	57.03	74.01	55.26
solvent	57.68	65.76	48.43
